# Role of m6A writers, erasers and readers in cancer

**DOI:** 10.1186/s40164-022-00298-7

**Published:** 2022-08-09

**Authors:** Zhen Fang, Wentong Mei, Chang Qu, Jiongdi Lu, Liang Shang, Feng Cao, Fei Li

**Affiliations:** 1 Department of General Surgery, Xuanwu Hospital, Capital Medical University, Beijing, China; 2 Department of Gastrointestinal Surgery, Shandong Provincial Hospital Affiliated to Shandong First Medical University, Jinan, Shandong China

**Keywords:** N6-methyladenosine (m6A), Cancer, Writers, Erasers, Readers

## Abstract

The N(6)-methyladenosine (m6A) modification is the most pervasive modification of human RNAs. In recent years, an increasing number of studies have suggested that m6A likely plays important roles in cancers. Many studies have demonstrated that m6A is involved in the biological functions of cancer cells, such as proliferation, invasion, metastasis, and drug resistance. In addition, m6A is closely related to the prognosis of cancer patients. In this review, we highlight recent advances in understanding the function of m6A in various cancers. We emphasize the importance of m6A to cancer progression and look forward to describe future research directions.

**Supplementary Information:**

The online version contains supplementary material available at 10.1186/s40164-022-00298-7.

## Introduction

**Fig. 1:**
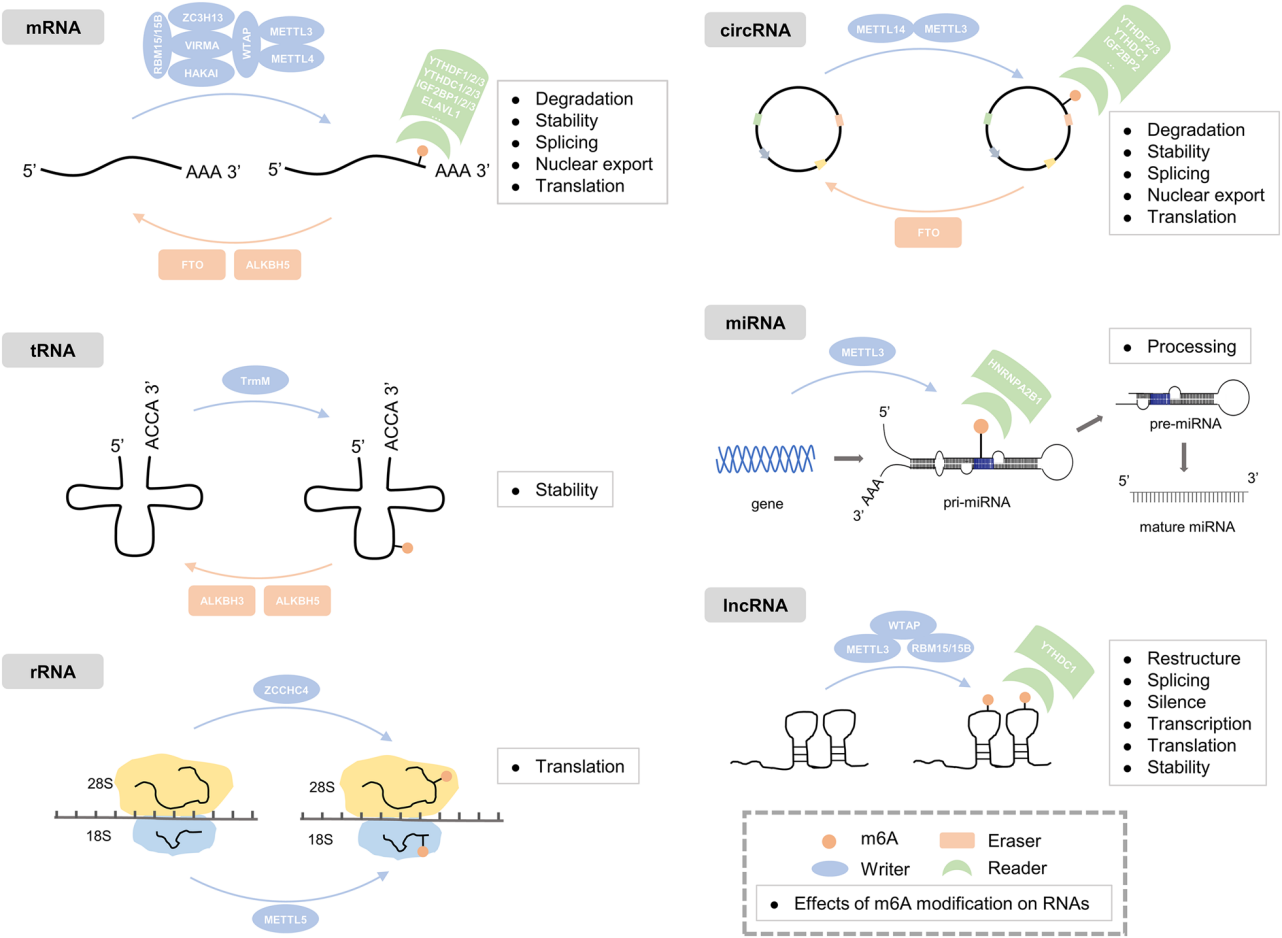
Effects of m6A modification in different types of RNA molecules (mRNA, tRNA, rRNA, circRNA, miRNA, lncRNA)

N6-methyladenosine (m6A) is the most prevalent, abundant and conserved posttranscriptional modification in eukaryotic RNAs and is deposited primarily within the RRACH consensus sequence [1, 2]. As one of the most common chemical modifications in eukaryotic RNAs, m6A exerts important effects on RNA stability, localization, translation, splicing, and transport [3–6]. m6A is widespread on mRNA, miRNA, lncRNA, circRNA, tRNA and other protein-coding and noncoding RNAs and has been a research focus in the field of epigenetics [7, 8]. (Fig. 1).

In the past ten years, with the development of new technologies, an increasing number of tools, such as next-generation sequencing, have been applied to research on m6A [9] (Table 1). m6A is involved in a variety of important cell processes, such as biological rhythms [10] and stem cell differentiation [11], and in a variety of diseases, including tumors [12–14] and obesity [15, 16]. Notably, m6A has been found to play an important role in the progression of human malignant tumors [8, 13]. Abnormal levels of m6A modification have been found in various tumors, and this disordered abundance is closely related to the progression, metastasis, drug resistance and prognosis of malignant tumors [17–20].

**Table 1:** m6A detection technologies

Technologies	References
m^6^A-Seq	[21]
MeRIP-seq	[22]
m^6^A-LAIC-seq	[6]
PA-m^6^A-Seq	[23]
miCLIP	[24]
m^6^A-CLIP	[25]
SCARLET	[26]
MAZTER-seq	[27]
RNAmod	[28]
FunDMDeep-m^6^A	[29]
Direct RNA sequencing	[30]
m^6^A-REF-seq	[31]

This review focuses on the progress of research into the mechanisms of m6A methylation, particularly with respect to regulatory proteins in various cancers. We also look forward and describe likely future m6A research trends.

## Writers, erasers, readers

**Fig. 2:**
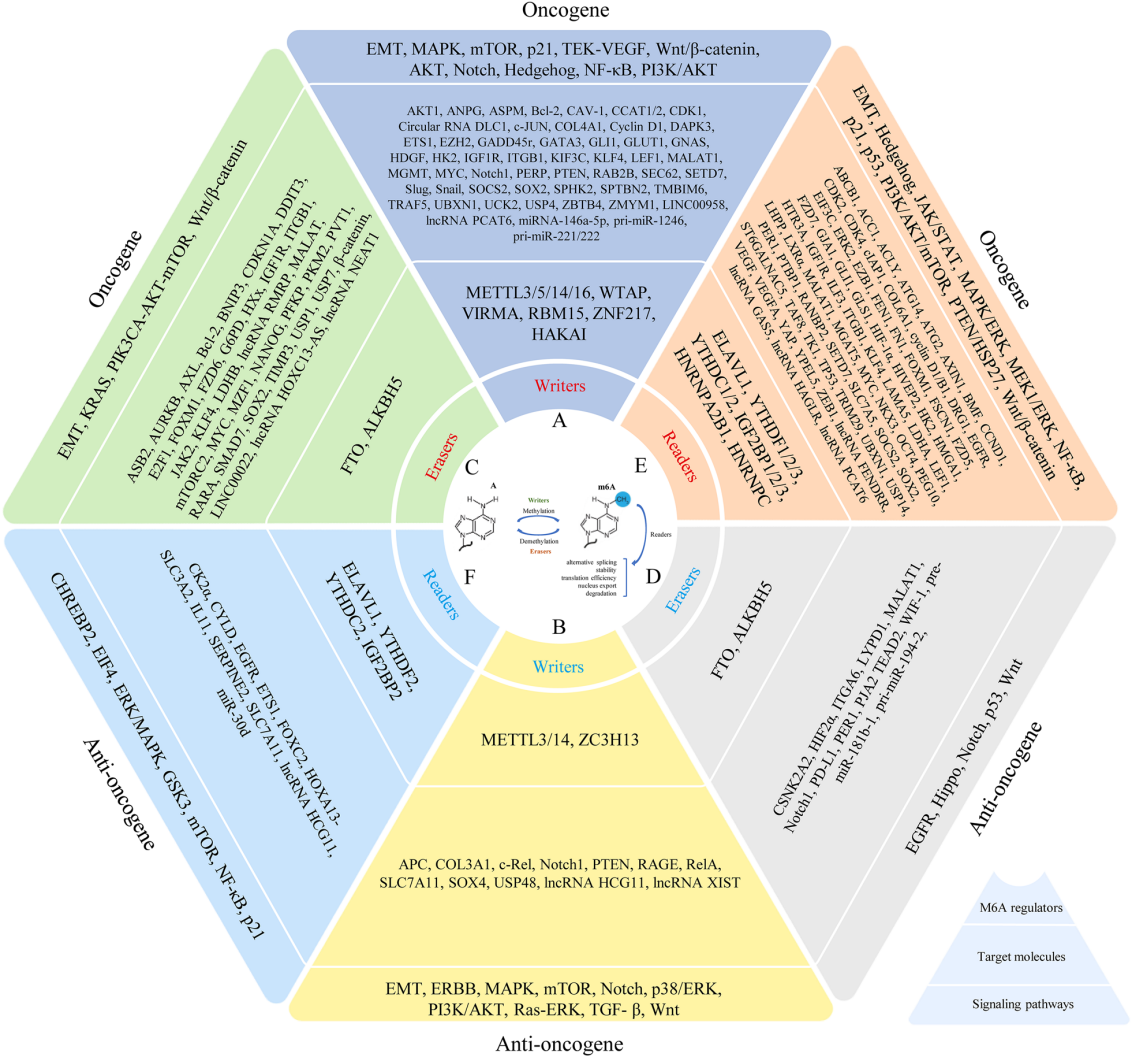
m6A regulators, as well as molecules and signaling pathways that can be regulated by m6A regulators. Red indicates regulators that play an oncogenic role, and blue indicates regulators that plat an anti-oncogenic role. **a**, **b** Writers. **c**, **d** Erasers. **e**, **f** Readers

There are three kinds of proteins that regulate m6A modification: writers, erasers, and readers [32]. Writers promote methylation and include METTL3, METTL5, METTL14, WTAP, RBM15, ZC3H13, and VIRMA. Erasers are demethylases and include FTO and ALKBH5. Readers are methylation reader proteins specific to m6A and include IGF2BP1/2/3, YTHDF1/2/3, and ELAVL1. These three types of regulatory proteins are often dysregulated in cancer. By regulating different downstream molecules and signaling pathways, they play roles in promoting cancer and/or suppressing cancer, affecting cancer progression and patient prognosis (Fig. 2).

### Writers

Methyltransferase-like 3 (METTL3), the only catalytic subunit of the m6A methyltransferase complex (MTC), was identified as the first m6A methyltransferase [17]. It can bind to approximately 22% of all m6A sites [33] and plays a dual role as an oncogene and a tumor suppressor in different tumors and, in some cases, in the same tumor [34, 35]. However, METTL3 is an oncogene in most tumors [36–40], although it has both carcinogenic and tumor-suppressing effects in colorectal cancer [41], breast cancer [42], prostate cancer [43], cervical cancer [35], and other cancers.

METTL5 is an m6A methyltransferase that functions works independently of the MTC to catalyze m6A of RNAs such as the U6 snRNA, 28S rRNA, and 18S rRNA [8]. Two m6A modification sites are located on mammalian ribosomal RNA, one at position 28S A4220 of the large subunit and the other at position 18S A1832 of the small subunit [44].

METTL14 is one of the important components of the MTC. METTL14 exerts carcinogenic and anticancer effects in different tumors. METTL14 can regulate the expression of PERP [45], USP48 [46], PTEN [47], and SOX4 [48] in a m6A-dependent manner and inhibit tumor proliferation, invasion, migration, metastasis and drug resistance. On the other hand, METTL14 can regulate the expression of miR-146A-5p [49] and the lncRNA OIP5-AS1 [50] and thus promote tumor development.

METTL16 was identified as a m6A methylase after METTL3, and the action of METTL16 is independent of the MTC. The substrates of METTL16 are considerably less abundant than those of METTL3 and include mainly U6 snRNA and S-adenosylmethionine (SAM) synthetase MAT2A [51].

WTAP is a regulatory subunit of the RNA methyltransferase complex, and it connects METTL3 to METTL14 and facilitates in the positioning of this dimer. Studies have found that WTAP affects the MAPK [52], AKT [52], Wnt [53, 54], and NF-κB [55] signaling pathways and promotes tumor progression by regulating the downstream targets EGR3 [56], HK2 [57], ETS1 [58], and CAV-1 [55]. KIAA1429 (VIRMA) participates in the formation of the MTC and serves as a scaffold. Studies have found that KIAA1429 induced m6A methylation on the 3’UTR of GATA3 pre-mRNA, which led to the degradation of GATA3 pre-mRNA and promoted the progression and metastasis of liver cancer [59].

RBM15 (RNA binding motif protein 15) belongs to the SPEN (split-end) family. It is located on chromosome 1p13.3. It can encode the RNA-binding protein RBM15, which is a protein homolog of RBM15B. RBM15/15B relies on WTAP to bind to the METTL3/METTL14 dimer, and knocking down RBM15/15B expression led to a significant decrease in the overall level of m6A, indicating that RBM15/15B is a functional component of the MTC [60, 61]. The role played RBM15 in the MTC with respect to tumor progression has been reported only for leukemia, liver cancer, and laryngeal cancer.

Zinc finger protein 217 (ZFP217) is a transcription factor with a conserved zinc finger structure that is highly expressed in a variety of cancers and is related to prognosis [62–69]. In 2016, research showed that ZNF217 inhibited the m6A methylation of KLF4 and NANOG mRNA, which was catalyzed by METTL3 and resulted in elevated KLF4 and NANOG protein levels, which in turn promoted the progression of breast cancer [70]. Zinc finger CCCH domain-containing protein 13 (ZC3H13) mainly promotes the binding of MTC with RNA [71]. ZC3H13 deletion led to a decrease in the overall m6A level of RNA, which was mainly attributed to reduced methylation of the 3’UTR in mRNA [72]. ZC3H13 has been shown to play a tumor-suppressing role, inhibiting the progression and metastasis of colorectal cancer and breast cancer by regulating the Ras-ERK and Wnt signaling pathways, respectively [73, 74].

A potential RING finger E3 ubiquitin ligase, Hakai is a member of the MTC, and it is the least studied molecule in the MTC. In 2021, Yan Dong and the Bawankar P’s team confirmed that Hakai is a core member of the m6A-modified protein family and an indispensable component of the MTC in Drosophila and human cells. However, no studies have shown that Hakai mediates tumor progression through m6A.

### Erasers

In 2011, Professor Chuan He first showed that fat mass and obesity-associated protein (FTO) can reverse m6A in vivo. FTO was thus the first m6A demethylase discovered, which led to an upsurge in basic m6A research [75]. In 2017, it was first reported that the FTO gene affected cancer progression [18]. Studies showed that FTO reduced the level of m6A on ASB2 and RARA mRNA transcripts, regulated the expression of targets, including ASB2 and RARA, inhibited the differentiation of AML cells induced by all-trans-retinoic acid (ATRA), and promoted the progression of AML [18]. FTO promoted tumor progression in liver cancer [76–78], lung cancer [79–83], breast cancer [84–86], cervical cancer [87–89], and colorectal cancer [90, 91]. However, FTO exerted a tumor-suppressing effect in kidney cancer [92–95], pancreatic cancer [96], thyroid cancer [97], and cholangiocarcinoma [98].

ALKBH5 was the second m6A demethylase discovered after FTO. ALKBH5 is involved in the biological progression of a variety of cancers, where it plays an important role [99–104]. PD-L1 mRNA is the direct target of the m6A mechanism, and the level of this mRNA is regulated by ALKBH5. Specifically, the deletion of ALKBH5 led to increased m6A abundance in the 3’UTR of PD-L1 mRNA, promoting mRNA degradation in a YTHDF2-dependent manner [103]. Therefore, ALKBH5 plays an important role in regulating the tumor immune microenvironment and mediating the effect of immunotherapy. ALKBH5 plays a dual role as a carcinogen and tumor suppressor in different cancers and, in certain cases, in the same type of cancer. ALKBH5 promotes cancer progression by regulating TIMP3 [105], FOXM1 [106], CDKN1A [107], JAK2 [108], FOXM1 [101, 109, 110], AURKB [111], G6PD [112], HBx [113], USP1 [114], NANOG [115], PVT1 [116], IGF1R [117] lncRNA NEAT1 [118, 119], and lncRNA RMRP [120] expression. In addition, ALKBH5 inhibited cancer progression by regulating PD-L1 [103], CK2α [121], LYPD1 [102], PER1 [122], WIF-1 [99], and lncRNA KCNK15-AS1 [123] expression.

### Readers

The YTH N6-methyladenosine RNA-binding protein (YTHDF) family consists of m6A readers. YTHDF family members located in the cytoplasm include YTHDF1, YTHDF2, and YTHDF3, which are also called DF1, DF2, and DF3, respectively. According to reports, these three DFs exhibit different functions. DF1 promotes the translation of mRNA, DF2 promotes the degradation of mRNA, and DF3 promotes translation and degradation of mRNA [124], but the mechanisms through which these three DFs perform different functions are unclear. Studies showed that YTHDF1/3 exhibited only carcinogenic effects in cancer [125–129], while YTHDF2 exerted both carcinogenic and anticarcinogenic effects [122, 130–132]. Therefore, the influence of the YTHFD family on the biological behavior of cancer and the reasons for the functional differences between family members need to be further studied.

The insulin-like growth factor-2 mRNA-binding protein (IGF2BP) family consists of unique m6A readers that, in contrast to YTH domain family proteins, do not promote mRNA degradation; in fact, they stabilize mRNA (such as MYC mRNA) [133]. The IGF2BP family includes IGF2BP1, IGF2BP2 and IGF2BP3. IGF2BP1 and IGF2BP3 are carcinoembryonic proteins that are produced by tumor and fetal tissues, but their expression is downregulated in adult tissues [8]. Recent studies revealed that IGF2BP1 bound to the 3’UTR m6A site of SOX2 mRNA and inhibited the degradation of SOX2 mRNA, which in turn led to the proliferation and metastasis of endometrial cancer cells [134]. IGF2BP protein family gene products have been found to be overexpressed in a variety of tumors and to regulate the stability of PEG10 [135], SOX2 [134], FSCN1 [7], MYC [7, 136], HMGA1 [137], YAP [138], LEF1 [139], FOXM1 [140], ABCB1 [141], CCND1 [142], VEGF [142], HIF1A [143], TMBIM6 [144], and lncRNA HAGLR [145] expression in an m6A-dependent manner to promote tumor progression.

Both YTHDC1/2 and YTHDF1/2/3 are mammalian m6A readers with a YTH domain. YTHDC1 regulates gene transcription through transposons [146], carRNA [147], chromatin modification [148], etc., and mRNA alternative splicing [149], stability [150] and subcellular localization [151] to regulate downstream target gene expression. Michael G Kharas’s team clarified the important role played YTHDC1 in AML and found that c-Myc was a key factor that mediated the functions of multiple m6A-related proteins in AML [152]. YTHDC2 is an RNA helicase whose helicase domain contributes to RNA binding and participates in the regulation of mRNA translation or degradation [153]. According to current research reports, YTHDC1/2 affected the progression of cancer by regulating CYLD [154], SLC7A11 [155], SLC3A2 [156], HOXA13 [156], and miR-30d [157].

Embryonic lethal abnormal vision-like protein 1 (ELAVL1), also known as human antigen R (HuR), is an RNA-binding protein that preferentially binds AU- or U-rich elements in the 3’UTR [158, 159]. ELAVL1 participates in a variety of tumor biological processes as an oncogene. Studies showed that ELAVL1 promoted the progression of liver cancer [160], lung cancer [161–163], colorectal cancer [164–166], gastric cancer [36], esophageal cancer [167], breast cancer [168, 169], prostate cancer [170, 171], and ovarian cancer [172]. However, few studies have investigated whether the effect of ELAVL1 on the expression of downstream molecules relies on m6A, and the role played by ELAVL1 in tumors is unclear.

The heterogeneous nuclear ribonucleoprotein (hnRNP) family consists of RNA-binding proteins that have been named hnRNPA1-U on the basis of their molecular weight [173]. The hnRNP complex includes at least 20 hnRNP proteins with complicated and diverse functions [173]. A large number of studies showed that hnRNPs were closely related to the occurrence and development of tumors. Recent studies showed that the interaction of the lncRNA MIR100HG with hnRNPA2B1 promoted m6A-dependent TCF7L2 mRNA stabilization and colorectal cancer progression [174]. HNRNPA2B1 recognizes the m6A site on ILF3 mRNA to stabilize ILF3 mRNA, leading to increased ILF3 expression and promoting the malignant progression of lymphoma [175].

## m6A and cancers

In recent years, many studies have proven that deregulation of m6A is closely related to various human cancers [8] (Table 2, Additional file 1: Table S1). These m6A regulators are described above. We explain the roles played by these molecules in tumor proliferation, invasion, migration, metastasis, drug resistance, and prognosis from the perspective of different cancers in the following subsections.

**Table 2:** Role of the modifier in cancer

Type of cancer	Role of the modifier	m6A(methylation) modifier
Breast cancer	Oncogene	METTL5, WTAP, VIRMA, ZNF217, FTO, ALKBH5, ELAVL1, YTHDF1/2/3, IGF2BP1/2/3, HNRNPs
	Tumor suppressor	ZC3H13, HAKAI
	Bivalent	METTL3/14
Lung Cancer	Oncogene	METTL3/5, WTAP, VIRMA, HAKAI, FTO, ELAVL1, YTHDF1, IGF2BP1/3, HNRNPs
	Tumor suppressor	METTL14, YTHDC2
	Bivalent	ALKBH5, YTHDF2, IGF2BP2
Prostate cancer	Oncogene	VIRMA, ZNF217, ELAVL1, YTHDF2, IGF2BP2, HNRNPs
	Tumor suppressor	FTO
	Bivalent	METTL3
Colorectal cancer	Oncogene	WTAP, ZNF217, HAKAI, ELAVL1, YTHDF1/2/3, YTHDC1/2, IGF2BP1/2/3, HNRNPs, FTO
	Tumor suppressor	METTL14, ZC3H13
	Bivalent	METTL3, ALKNH5
Gastric cancer	Oncogene	METTL3/16, WTAP, VIRMA, ZNF217, ALKBH5, ELAVL1, YTHDF1, IGF2BP1/2/3, HNRNPs
	Tumor suppressor	METTL14, YTHDF2
	Bivalent	FTO
Liver cancer	Oncogene	METTL3, WTAP, VIRMA, ZNF217, FTO, YTHDF1/3, YTHDC2, IGF2BP1/2/3, HNRNPs
	Tumor suppressor	METTL14
	Bivalent	ALKBH5, ELAVL1, YTHDF2
Cervical cancer/ endometrial cancer/ ovarian cancer	Oncogene	ZNF217, WTAP, FTO, ALKBH5, YTHDF1, IGF2BP1/2/3, HNRNPs, ELAVL1, YTHDF2
	Bivalent	METTL3, YTHDF2
Esophageal cancer	Oncogene	METTL3, WTAP, FTO, ELAVL1, IGF2BP1/2/3, HNRNPs
	Bivalent	ALKBH5
Thyroid cancer	Oncogene	METTL14, IGF2BP1/2/3
	Tumor suppressor	FTO
	Bivalent	METTL3
Bladder cancer	Oncogene	METTL3, WTAP, YTHDF2, IGF2BP1
	Tumor suppressor	METTL14, ALKBH5
	Bivalent	FTO
Pancreatic cancer	Oncogene	METTL3/14, WTAP, YTHDF2, IGF2BP2/3, HNRNPs
	Tumor suppressor	FTO, ALKBH5, YTHDC1
Leukaemia	Oncogene	METTL3/14, WTAP, FTO, ALKBH5, YTHDF2, IGF2BP1/2/3, RBM15
Kidney cancer	Oncogene	WTAP, IGF2BP1/3, HNRNPs
	Tumor suppressor	METTL14, FTO, YTHDF2
	Bivalent	ALKBH5
Melanoma	Oncogene	METTL3, FTO, ALKBH5, ELAVL1, YTHDF1/2, IGF2BP1/2/3, HNRNPs
Head and neck cancer	Oncogene	METTL3, FTO, ALKBH5, ELAVL1, YTHDF1, IGF2BP1/2//3, HNRNPs
	Bivalent	YTHDC2
Glioblastoma	Oncogene	METTL3, ZNF217, ALKBH5, ELAVL1, YTHDF1/2, IGF2BP1/2/3
Osteosarcoma	Oncogene	METTL14, ZNF217, ELAVL1, IGF2BP1
	Tumor suppressor	YTHDF2
	Bivalent	ALKBH5
Cholangiocarcinoma/ gallbladder cancer	Oncogene	IGF2BP1/2
	Tumor suppressor	FTO, ALKBH5
Retinoblastoma	Oncogene	METTL3, IGF2BP1
lymphomas	Oncogene	METTL3/14, WTAP, RBM15, FTO, ALKBH5, YTHDF2, IGF2BP1/2/3
Rhabdomyosarcoma	Oncogene	IGF2BP1
Seminoma	Oncogene	METTL3
Thymic epithelial cancer	Oncogene	METTL3

See Additional file 1: Table S1 for detailed references

### Breast cancer

Breast cancer is a major cause of morbidity and mortality in women worldwide, accounting for 11.7% of all cancer cases, and the mortality rate ranks fifth among cancers [176]. Many studies have been carried out to analyze the mechanism of the m6A effect on breast cancer. Writers, erasers, and readers mainly play cancer-promoting roles, participating in cancer cell proliferation, invasion, metastasis and drug resistance [177–180]. KIAA1429 regulates the expression of CDK1 in an m6A-dependent manner and exerts a carcinogenic effect on breast cancer [181]. YTHDF3 promotes brain epithelial cell adhesion, invasion and tumor cell angiogenesis, which is closely related to breast cancer brain metastasis [128]. METTL3 accelerates the maturation of pri-microRNA221-3p in a m6A-dependent manner, leading to adriamycin resistance in breast cancer cells [182]. In addition, a few studies have suggested that certain writers (METTL3 [42], METTL14 [74], and ZC3H13 [74]) exert a tumor-suppressing effect. For example, Yuee Teng’s team found that METTL3 downregulated the expression of COL3A1 by increasing the m6A abundance on COL3A1 mRNA and thus inhibited the metastasis of triple-negative breast cancer cells [42].

### Lung cancer

Lung cancer is the second most common cancer, with an estimated 2.2 million new cancer cases and 1.8 million deaths each year, accounting for approximately one-tenth (11.4%) of diagnosed cancers and one-fifth (18.0%) of cancer-related deaths [176]. Writers play a cancer-promoting role in lung cancer and are significantly related to poor prognosis, except for METTL14 [183–192]. METTL3/YTHDF2 reduces the expression of ZBTB4 mRNA in a m6A-dependent manner, enhances the expression of EZH2, induces the EMT, and promotes the proliferation and metastasis of lung cancer [186]. However, ALKBH5 inhibits the growth and metastasis of NSCLC by reducing YTHDF-mediated YAP expression and inhibiting miR-107/LATS2-mediated (HuR-dependent) YAP activity [193]. In addition, many m6A readers have been found to be involved in the occurrence and development of lung cancer [20, 127, 194, 195]. YTHDF1 promotes the translation of cyclin B1 mRNA in an m6A-dependent manner, thereby promoting KRAS/TP53-mut LUAD proliferation and leading to poor prognosis [195]. YTHDC2 exerts an antitumor effect on lung cancer [154–156, 196]. M6A plays an important role in the proliferation [82, 106, 120], invasion [80, 83, 106], metastasis [79, 193], drug resistance [197, 198] and prognosis [191] of lung cancer and may become a new molecular therapeutic target.

### Prostate cancer

Prostate cancer is the second most common cancer in men and the fifth leading cause of cancer deaths, with approximately 1.4 million new cases and 375,000 deaths each year [176]. Studies showed that METTL3 regulates LEF1 [199], KIF3C [200], USP4 [201], GLI1 [202], ITGB1 [170], IGF1R [203], and lncRNA PCAT6 [203] expression in an m6A-dependent manner to promote prostate cancer malignant progression. One study showed that knocking out METTL3 drives prostate cancer cell resistance to androgen receptor antagonists; hence, the change in m6A abundance may be a mechanism underlying treatment resistance in metastatic prostate cancer [43]. To date, only study has reported the role played by erasers in the development and progression of prostate cancer [204]. The m6A demethylase FTO inhibits the invasion and migration of prostate cancer cells by regulating the total m6A level [204].

### Colorectal cancer

Colorectal cancer (CRC) has the third highest incidence, as measured by total cases, and the second highest mortality among total cancer deaths, with more than 1.9 million new cancer cases and 935,000 deaths estimated to occur yearly [176]. In CRC, all readers and most writers and erasers show cancer-promoting effects, except for METTL3 [41], METTL14 [205, 206] and ALKBH5 [207]. Professor Zhou Yang’s team found that after knocking down METTL3, the reduction in translation efficiency of the important EMT regulators Snail and HIF-1α depends on m6A modification, and the reduced activity of these regulators significantly inhibits the proliferation and clone formation of CRC cells [208]. However, an article reported that METTL3 inhibited the proliferation, migration and invasion of CRC cells by regulating the p38/ERK pathway (activating p-p38 and p-ERK) [41]. The reason for these contradictory findings may be explained by METTL3 playing different roles in the regulation of different pathways, but the METTL3-regulated pathways that contribute to a tumor-suppressing effect remain unknown.

### Gastric cancer

Gastric cancer is a common malignant tumor of the digestive system, and it is responsible for more than 1 million new cases and an estimated 769,000 deaths every year [176, 209]. Studies have found that m6A is involved in the regulation of gastric cancer cell proliferation [210–213], invasion [214], migration [215], metastasis [34, 36, 216, 217] and drug resistance [218]. M6A modifiers are important biomarkers for early gastric cancer diagnosis, prognosis and therapy predictions [217, 219, 220]. METTL3 enhances the stability of ZMYM1 as facilitated by HuR via m6A and activates the EMT to promote the metastasis of gastric cancer [36]. Most m6A modifiers play a role in promoting gastric cancer, and only a few studies have reported m6A modifiers that play a tumor-suppressing role in gastric cancer. METTL14 increases m6A of PTEN mRNA, stabilizes PTEN mRNA, increases protein expression, and inhibits the growth and metastasis of gastric cancer [47]. METTL14 also inhibits the proliferation and invasion of gastric cancer cells by inhibiting PI3K/AKT/mTOR pathway activation and the EMT [221]. YTHDF2 negatively regulates the expression of FOXC2 through m6A and inhibits the proliferation, invasion and migration of gastric cancer cells [132]. According to recent research results, m6A plays an important role in the progression of gastric cancer.

### Liver cancer

Primary liver cancer is the sixth most common cancer in the world and the third leading cause of cancer deaths; approximately 906,000 new cases and 830,000 deaths are reported each year [176]. Many studies on m6A regulators in liver cancer have been performed, and the main role of m6A has been shown to be cancer promotion. METTL3 regulates the expression of the downstream targets ASPM [222] and SOCS2 [40] in a m6A-dependent manner and promotes the proliferation and metastasis of liver cancer. KIAA1429 regulates circular RNA DLC1 to promote cancer through m6A mediation [223]. METTL14 plays a tumor-suppressing effect in liver cancer and is the only m6A modifier that suppresses liver cancer tumorigenesis [46, 224–227]. The m6A demethylase FTO and ALKBH5 play roles in promoting liver cancer progression. FTO promotes the occurrence of liver cancer by mediating the demethylation of PKM2 mRNA [76]. ALKBH5 catalyzes the demethylation of m6A of the HBx mRNA, stabilizes and promotes the expression of the HBx mRNA, and promotes hepatocellular carcinogenesis [113]. However, a study found that ALKBH5-mediated m6A demethylation resulted in the posttranscriptional inhibition of LYPD1 expression, which in turn may have inhibited the progression of liver cancer [102].

The m6A readers in the YTHDF protein family all play a role in promoting liver cancer progression, except for YTHDF2 [131, 224, 228]. YTHDF1 promotes the progression of liver cancer by regulating FZD5 [229], ATG2A/14 [126], and PI3K/AKT/mTOR signaling activity [230] and the EMT [231]. The IGF2BP protein family of m6A readers all play a role in promoting liver cancer progression. The downstream targets regulated by these family members include c-MYC [232, 233], MGAT5 [234], GLI1 [235], IGF1R [236], FEN1 [237], and TRAF5 [238]. HNRNPA2B1 can promote the proliferation and invasion of liver cancer cells, but whether it depends on m6A is unclear.

### Cervical cancer

Cervical cancer is the fourth most common cancer and the fourth leading cause of cancer death in women [176]. In 2020, there were an estimated 604,000 new cases and 342,000 deaths worldwide [176]. Recent research results have indicated that m6A regulators play a role in promoting cervical cancer [87–89, 239–247]. Only one study found that METTL3 downregulated the expression of RAGE in cervical cancer cells, inhibited cell viability, increased cell apoptosis, and enhanced the sensitivity of these cells to cisplatin therapy [35]. The downstream targets of m6A regulators in cervical cancer are HK2 [239], the lncRNA FOXD2-AS1 [241], RAB2B [242], RAGE [35], E2F1 [87], MYC [87], β-catenin [88], the lncRNA HOXC12-AS [89], RANBP2 [245], and FOXM1 [140].

### Endometrial cancer

Endometrial cancer is one of the most common female reproductive system tumors, with approximately 200,000 new cases diagnosed each year, and is the third most common gynecological malignancy that causes death (after ovarian cancer and cervical cancer) [248]. Few studies have been directed to m6A regulators in endometrial cancer, and the results of these studies have indicated that these regulators mainly promote cancer. FTO demethylates m6A of HOXB13 mRNA and promotes endometrial cancer metastasis by activating the WNT signaling pathway [249]. YTHDF2 recognizes m6A on the lncRNA FENDRR to promote the lncRNA degradation, thereby increasing the expression of SOX4 to promote the proliferation and inhibit the apoptosis of endometrial cancer cells [250]. IGF2BP1 recognizes the m6A site on PEG10 and SOX2 mRNAs and increases the expression of these mRNAs by enhancing their stability, promoting the malignant progression of endometrial cancer [134, 135].

### Ovarian cancer

The incidence of ovarian cancer ranks third among gynecological malignancies, with 230,000 new cases diagnosed each year [251]. However, the mortality rate of ovarian cancer greatly exceeds that of cervical cancer or endometrial cancer, ranking first in gynecological cancer deaths [251]. The results of recent research have indicated that m6A regulators in ovarian cancer all exert cancer-promoting effects. METTL3-mediated miR-126-5p maturation promotes the progression of ovarian cancer through the PI3K/Akt/mTOR pathway mediated by PTEN [252]. METTL3 also inhibits the expression of CCNG2 by promoting pri-microRNA-1246 maturation, thereby promoting the occurrence and metastasis of ovarian cancer [253]. ALKBH5 activates the JAK2/STAT3 signaling pathway by mediating JAK2 m6A demethylation to promote cisplatin resistance in ovarian cancer [108]. YTHDF1 promotes the translation of TRIM29 mRNA by recognizing the 3’UTR m6A site and thus promotes the progression of ovarian cancer [254]. YTHDF2 significantly downregulates the level of m6A and promotes the proliferation and migration of ovarian cancer cells [255].

### Esophageal cancer

The incidence and mortality of esophageal cancer rank seventh and sixth, respectively, with approximately 604,000 new cases and 544,000 deaths reported each year [176]. Studies have found that the m6A regulators in esophageal cancer all play a cancer-promoting role, except for ALKBH5 [100, 256]. The downstream targets that the m6A writer METTL3 regulates in esophageal cancer are GLS2 [257], p21 [258], Wnt/β-catenin [38, 259], AKT [259], the EMT [259], and the Notch [260] signaling pathway. ALKBH5 plays dual roles in promoting and suppressing tumor growth in esophageal cancer. Knocking down ALKBH5 upregulates m6A level on CDKN1A(p21) mRNA, increases the stability of p21 mRNA, and promotes the proliferation of esophageal cancer cells by regulating cell cycle progression [107]. However, a study showed that ALKBH5 inhibits the malignant behavior of esophageal cancer by indirectly regulating the Hippo signaling pathway [100].

In addition, the IGF2BP protein family plays an important role in the proliferation, invasion, migration and metastasis of esophageal cancer and can be used as biomarkers for predicting prognosis. The downstream targets regulated by IGF2BP protein family members are UHRF2 [261], TK1 [262], and HTR3A [263]. High protein expression of IGF2BP predicts poor prognosis in patients with esophageal cancer [264, 265]. HNRNPC enhances the stability of ZEB1 and ZEB2 mRNA and promotes the development of esophageal squamous cell carcinoma [266]. High expression of HNRNPA2B1 has been associated with a low survival rate in esophageal cancer [267].

### Thyroid cancer

In 2020, the global incidence of thyroid cancer was 586,000, and the incidence in women was threefold greater than that in men [176]. Thyroid cancer is the most common endocrine cancer, and its incidence is increasing globally, but the cause for this increase is unclear [176]. Studies showed that the upregulation of miR-222-3p induced by METTL3 inhibits STK4 activity and promotes the malignant behavior of thyroid cancer cells [268]. However, another study found that METTL3 cooperates with YTHDF2 to regulate downstream c-Rel and RelA, participates in the inactivation of the NF-κB pathway, and plays a key tumor-suppressing role in papillary thyroid cancer [269]. IGF2BP2 can regulate the expression of MYC [136], lncRNA HAGLR [145], and IGF2 [270] in a m6A-dependent manner and promote the progression of thyroid cancer.

### Bladder cancer

Bladder cancer is the tenth most common cancer in the world, with approximately 573,000 new cases and 213,000 deaths reported each year, and is more common in men than women [176]. Recent studies showed that METTL3-mediated m6A hypermethylation promotes the progression of bladder cancer by regulating SETD7/KLF4 mRNA expression [271], pri-mrR221/222 maturation [37] and AFE4/NF-κB/MYC signaling network activation [272]. METTL14 inhibits the occurrence and progression of bladder cancer by regulating the EMT [273] and the Notch [274] signaling pathway. ALKBH5 can reduce the stability of CK2α mRNA in a m6A-dependent manner, significantly inhibiting the proliferation of bladder cancer cells and sensitizing bladder cancer cells to cisplatin in vivo and in vitro [121]. M6A readers (YTHDF2, IGF2BP1, and IGF2BP3) promote the progression of bladder cancer by stabilizing SETD7 [271], KLF4 [271], FSCN1 [7], and MYC [7] mRNA and regulating JAK/STAT [275] signaling pathway activation.

### Pancreatic cancer

Pancreatic adenocarcinoma is a malignancy with an extremely poor prognosis, high mortality and short survival. In 2020, the number of deaths from pancreatic cancer (466,000) was almost as great as the number of patients (496,000), and it is the seventh leading cause of cancer deaths [176]. The role played by m6A in pancreatic cancer is gradually being discovered. m6A has been found to be closely related to the occurrence [96, 276], progression [45, 54], drug resistance [99, 277–279] and prognosis [280, 281] of pancreatic cancer. Studies have found that the METTL3-miR-25-3p-PHLPP2-AKT signaling axis may be related to the occurrence and development of smoking-related pancreatic cancer [282]. Inhibition of METTL14 expression can significantly increase the sensitivity of drug-resistant pancreatic cancer cells to gemcitabine [278] and cisplatin [277]. The m6A erasers FTO and ALKBH5 play roles in promoting pancreatic cancer. FTO inhibits the occurrence of pancreatic cancer by reducing the methylation level of PJA2 mRNA and inhibiting Wnt signaling pathway activation [96]. ALKBH5 regulates WIF-1 [99], PER1 [122], and lncRNA KCNK15-AS1 [123] expression in an m6A-dependent manner and inhibit the occurrence and progression of pancreatic cancer tumors.

### Leukemia

Leukemia is a heterogeneous malignant disease characterized by the accumulation of clonal and undifferentiated hematopoietic cells in the bone marrow and blood [283]. Its incidence is increasing every year, and improving treatment effectiveness remains a great challenge [283, 284]. Recent studies have found that m6A regulators all play roles in promoting leukemia. In 2017, Professor Tony Kouzaridesd found that overexpression of METTL3 promoted the development of acute myeloid leukemia (AML) [39]. The research team identified, for the first time, a small-molecule inhibitor of the m6A methylase METTL3 in the body, STM2457, and confirmed that this inhibitor effectively inhibits the development of acute myeloid leukemia (AML) [39]. FTO and ALKBH5 are also involved in the occurrence and promotion of leukemia. FTO regulates the translation of PFKP, LDHB, ASB2, and RARA mRNA in a m6A-dependent manner to promote the occurrence of leukemia [18, 285]. ALKBH5 also plays a key role in promoting the occurrence of leukemia by regulating the activity of key targets (such as TACC3 and USP1) [114, 286].

### Kidney cancer

Kidney cancer is among the ten most prevalent cancers, with approximately 430,000 new cases and 180,000 deaths reported every year [176]. Kidney cancer is difficult to detect and treat, and little is known about it [287]. Few studies have been directed to the mechanism of m6A action in renal cancer. Studies showed that the m6A writer METTL14 regulates the expression of PTEN in a m6A-dependent manner and inhibits the progression of clear cell renal cell carcinoma (ccRCC) [288]. FTO may be involved in the regulation of ccRCC by regulating the downstream target BRD9 [94].

### Melanoma

Melanoma is a highly malignant tumor derived from melanocytes. Although it is mostly likely to affect skin mucous membranes and internal organs can be affected. After the introduction of new therapies, including immune checkpoint inhibitors and targeted treatment of metastatic melanoma, the mortality rate of melanoma in the United States has decreased markedly, by approximately 6.4% every year, but some patients who cannot benefit from immunotherapy [176, 289]. The role played by m6A in melanoma is not fully understood, and only a few research results have been reported. A study found that METTL3 induced UCK2 m6A hypermethylation and promoted the metastasis of melanoma cells through the WNT/β-catenin pathway [290]. METTL3 may be involved in the proliferation, invasion, migration and resistance of melanoma cells [291, 292]. ALKBH5 increases the stability and expression of FOXM1 mRNA via m6A demethylation and induce the epithelial-mesenchymal transition (EMT) to promote melanoma metastasis [110]. FTO promotes the growth of melanoma and reduces the response to anti-PD-1 blocking immunotherapy [293].

### Head and neck cancer

Head and neck cancer is a squamous cell carcinoma that originates from the mucosal surface of the oral cavity, nasal cavity, pharynx, larynx, or nasopharyngeal cavity. According to 2018 data, head and neck cancer is the seventh most common cancer in the world, with 890,000 new cases and 450,000 deaths [294]. METTL3 modulates m6A of CDC25B and promotes the malignant progression of head and neck squamous cell carcinoma [295]. METTL3 regulates the m6A levels on EZH2, tankyrase and snail and promotes the progression of nasopharyngeal carcinoma [296–298]. METTL3 may regulate the expression of PRMT5, PD-L1 and c-MYC through m6A to promote the progression of oral squamous cell carcinoma [299, 300]. YTHDC2 physically binds to insulin-like growth factor 1 receptor (IGF1R) mRNA to promote the translation of IGF1R mRNA, which in turn activates the IGF1-AKT/S6 signaling pathway and promotes radiotherapy resistance in nasopharyngeal carcinoma cells [301]. RBM15-mediated m6A modification of TMBIM6 mRNA enhances the stability of TMBIM6 mRNA in an IGF2BP3-dependent manner and promotes the progression of laryngeal squamous cell carcinoma [144].

### Glioblastoma

Glioblastoma (GBM) is a rare tumor and one of the most challenging malignant tumors to treat. The prognosis and quality of life of patients are very poor [302]. Studies have found that m6A regulators play roles in promoting malignant gliomas and are closely related to glioma cell proliferation [303–305], invasion [304, 305], metastasis [306], drug resistance [307] and prognosis [308]. METTL3 regulates MGMT, ANPG, COL4A1, MALAT1, and UBXN1 expression in an m6A-dependent manner and promotes the progression of malignant glioma [303–305, 307]. ALKBH5 regulates FOXM1, G6PD, SOX2, and AKT2 expression in an m6A-dependent manner to promote cancer cell proliferation, invasion and drug resistance [101, 112, 309–311]. The YTHDF protein family regulates LXRA [312], HIVEP2 [312], MYC [313], VEGFA [313], and UBXN1 [305] expression in an m6A-dependent manner and promotes the occurrence, metastasis and drug resistance of malignant gliomas.

### Osteosarcoma

Osteosarcoma is the most common primary bone cancer in children and young adults. It is a very rare cancer, and there are approximately 400 newly diagnosed cases in children and young adults in the United States each year [314]. There are few studies on m6A in osteosarcoma, and the conclusions reported have been inconsistent. ALKBH5 inhibits the progression of osteosarcoma through m6A-dependent epigenetic silencing of the premiR-181b-1/YAP signaling axis [315]. Another study found that ALKBH5 mediates the upregulation of PVT1 expression through m6A and promotes the proliferation of osteosarcoma cells [116]. METTL3 and ELAVL1 induce the upregulation of DRG1 expression in an m6A-dependent manner and promote the occurrence of osteosarcoma [316].

### Other cancers

Studies on cholangiocarcinoma have found that PD-L1 mRNA is the direct target of m6A, and this modification level is regulated by ALKBH5. The deletion of ALKBH5 increased m6A abundance on the 3’UTR of PD-L1 mRNA. ALKBH5 plays a role in regulating the tumor immune microenvironment and the effect of immunotherapy [103]. In retinoblastoma, the m6A methyltransferase METTL3 promotes retinoblastoma progression through the PI3K/AKT/mTOR pathway [317]. In rhabdomyosarcomas, IGF2BP1 directly binds to cIAP1 mRNA and mediates its translation, regulating rhabdomyosarcoma cell death and drug resistance [318]. In seminoma, METTL3 regulates autophagy and sensitivity to cisplatin by targeting ATG5 [319]. In thymic epithelial tumors, METTL3 promotes cell proliferation by controlling the expression of c-MYC, thereby causing carcinogenesis [320].

## Current status and future perspectives

As the most common modification on eukaryotic RNA, m6A is a star in cancer research. m6A has been reported in many cancers and confirmed to be involved in biological processes of tumors. Although there are some inconsistencies in the literature that require further detailed research to resolve, considerable evidence supports the importance of m6A in regulating the malignant progression of a variety of cancers. In the previous section, we summarized the molecular mechanisms of the three types of m6A regulatory proteins in cancer progression and the biological roles played by m6A regulatory proteins in different cancers. However, the importance of m6A in the occurrence and development of cancer is still unclear, and whether it can completely turn off the “switch” that causes cancer is unknown. Many problems and challenges remain to be solved in the future.

Some research findings suggest that m6A is a double-edged sword in cancer; that is, m6A regulators play different roles in different cancers. Moreover, one m6A regulator can act as both a tumor promoter and tumor suppressor in the same cancer [34, 35, 42, 216]. We need to determine why an m6A regulator plays different roles in different cancers or in the same cancer. Recent research results revealed that m6A-modified regulators target different downstream molecules and signaling pathways, which may among the reasons for their different biological effects. In addition, tumors show obvious heterogeneity. In different patients or different cancer cell subgroups of the same patient, an m6A regulatory factor may regulate different downstream targets, leading to two effects: cancer promotion and cancer suppression. We also need to pay attention to m6A-related regulators that not only play biological roles by regulating m6A in vivo but also play other important roles; some of these regulators may be responsible for certain contradictory results.

In recent years, with the continuous deepening of research, the roles played by m6A and its regulatory factors in the occurrence and development of cancer have become increasingly obvious. However, most of the recent studies on m6Ahas been basic molecular research, and whether m6A can be targeted for cancer treatment remains to be determined. In addition, the results of analysis based on data from public databases (such as the TCGA and GEO) cannot be used as sufficient evidence that m6A is related to tumors. Therefore, a large number of basic and clinical studies need to be carried out. In 2019, Professor Caiguang Yang’s team reported that an FTO inhibitor, FB23-2, significantly inhibited the proliferation of human acute myeloid leukemia (AML) cell lines and primary AML blasts in vitro [321]. In 2020, Professor Jianjun Chen’s team discovered two small-molecule compounds, CS1 (bisantrene) and CS2 (brequinar), which are powerful FTO inhibitors, that not only reduced the number of leukemia stem cells but also significantly inhibited the immune escape of leukemia cells [322]. In April 2021, a study at the University of Cambridge in the United Kingdom reported the first small-molecule inhibitor of the m6A methylase METTL3 that is active in the body—STM2457 [39]. These research results indicate that encouraging steps have been made toward evaluating the clinical application and potential clinical significance of m6A.

Innovations such as high-throughput sequencing and mass spectrometry technology have facilitated the progress of m6A research, which has been helpful for gaining a more comprehensive and in-depth understanding of cell development and tumor formation [323]. Although the number of studies on m6A modification has increased, the technology for detecting m6A is still characterized by high cost, low precision, and insufficient sensitivity. In the future, it is necessary to vigorously promote the innovation and development of analytical technology, continuously improve detection accuracy and sensitivity, and reduce detection costs at the same time.

## Conclusions

In this review, we summarize the biological characteristics of m6A writers, readers, and erasers in cancers. Writers can catalyze m6A modifications on RNA, while erasers can remove these modifications. Readers affect RNA splicing, export, degradation, translation and other biological processes by recognizing m6A methylation. Studies have found that these m6A regulators play an important role in regulating the occurrence, development, metastasis, drug resistance and other biological processes in cancer. However, the specific molecular mechanism by which m6A methylation affects tumor biological behavior is still unclear.

It is undeniable that m6A shows good application prospects for cancer treatment. In the future, m6A may become a novel diagnostic or treatment target for cancer. However, this is a tortuous and lengthy process that requires many basic and clinical research studies as well as technological advances. This review comprehensively summarizes the recent research progress on the m6A methylation modification in human cancer and provides a theoretical basis and direction for future research on m6A in the field of cancer.

## Supplementary Information

**Additional file 1: Table S1.** Role of the modifier in cancer. (Including references).

## Data Availability

Not applicable.

## Abbreviations

ABCB1: ATP binding cassette subfamily B member 1
ACLY: ATP citrate lyase
AKT: AKT serine/threonine kinase
ALKBH5: AlkB homolog 5
AML: Acute myelocytic leukemia
ARHGDIA: Rho GDP dissociation inhibitor alpha
ASB2: Ankyrin repeat and SOCS box containing 2
ASPM: Assembly factor for spindle microtubules
ATG2A/5/14: Autophagy related 2A5//14
ATM: ATM serine/threonine kinase
AURKB: Aurora kinase B
BCL-2: BCL2 apoptosis regulator
BNIP3: BCL2 interacting protein 3
BRD9: Bromodomain containing 9
CAV-1: Caveolin 1
CCAT1/2: Colon cancer associated transcript 1/2
CCND1: Cyclin D1
CCNG2: Cyclin G2
ccRCC: Clear cell renal cell carcinoma
CDC25C: Cell division cycle 25C
CDK1/4: Cyclin dependent kinase 1/4
CDKN1A: Cyclin dependent kinase inhibitor 1A
CHK2: Checkpoint kinase 2
c-Jun: Jun proto-oncogene
CK2α: Casein Kinase 2 Alpha 1
COL3A1: Collagen type III alpha 1 chain
COL4A1: Collagen type IV alpha 1 chain
COL6A1: Collagen type VI alpha 1 chain
CRC: Colorectal cancer
c-Rel: REL proto-oncogene
CYLD: CYLD lysine 63 deubiquitinase
DANCR: Differentiation antagonizing non-protein coding RNA
DDX3: DEAD-box helicase 3 X-linked
DLC1: DLC1 Rho GTPase activating protein
DRG1: Developmentally regulated GTP binding protein 1
E2F1: E2F transcription factor 1
EGFR: Epidermal growth factor receptor
EGR3: Early growth response 3
eIF3: Eukaryotic translation initiation factor 3
ELAVL1/HuR: ELAV like RNA binding protein 1
EMT: Epithelial-mesenchymal transition
ENO2: Enolase 2
ETS1: ETS proto-oncogene 1
FBW7: F-box and WD repeat domain containing 7
FEN1: Flap structure-specific endonuclease 1
FENDRR: FOXF1 adjacent non-coding developmental regulatory RNA
FN1: Fibronectin 1
FOXC2: Forkhead box C2
FOXM1: Forkhead box M1
FSCN1: Fascin actin-bundling protein 1
FTO: FTO alpha-ketoglutarate dependent dioxygenase
FZD5/9: Frizzled class receptor 5/9
G6PD: Glucose-6-phosphate dehydrogenase
GAS5: Growth arrest specific 5
GATA3: GATA binding protein 3
GBM: Glioblastoma
GEO: Gene expression omnibus
GLI1: GLI family zinc finger 1
GLS1/2: Glutaminase1/2
GLS2: Glutaminase 2
GLUT1: Solute carrier family 2 member 1
GLUT4: Solute carrier family 2 member 4
HAGLR: HOXD antisense growth-associated long non-coding RNA
HAKAI: Cbl proto-oncogene like 1
HBx: Late endosomal/lysosomal adaptor, MAPK and MTOR activator 5
HCC: Hepatocellular carcinoma
HCG11: HLA complex group 11
HDGF: Heparin binding growth factor
HIF-1α: Hypoxia inducible factor 1 subunit alpha
HIVEP2: HIVEP zinc finger 2
HK2: Hexokinase 2
HMGA1: High mobility group AT-Hook 1
hnRNPs: Heterogeneous nuclear ribonucleoprotein
HOXA1: Homeobox A1
HTR3A: 5-Hydroxytryptamine receptor 3A
IDH: Isocitrate dehydrogenase (NADP( +)) 1
IGF1R: Insulin-like growth factor 1 receptor
IGF2BP1/2/3: Insulin like growth factor 2 MRNA binding protein 1/2/3
IL11: Interleukin 11
ILF3: Interleukin enhancer binding factor 3
IRS1: Insulin receptor substrate 1
ITGB1: Integrin subunit beta 1
JAK2: Janus kinase 2
KDM4C: Lysine demethylase 4C
KIAA1429/VIRMA: Vir like M6A methyltransferase associated
KIF3C: Kinesin family member 3C
KLF4: Kruppel like factor 4
KRAS: KRAS proto-oncogene
LAMA5: Laminin subunit alpha 5
LDHB: Lactate dehydrogenase B
LEF1: Lymphoid enhancer binding factor 1
LHPP: Phospholysine phosphohistidine inorganic pyrophosphate phosphatase
lncRNA: Long non-coding RNA
LUAD: Lung adenocarcinoma
LXRA: Nuclear receptor subfamily 1 group H member 3
LYPD1: LY6/PLAUR domain containing 1
MALAT1: Metastasis associated lung adenocarcinoma transcript 1
MAPK: Mitogen-activated protein kinase
MAT2A: Methionine adenosyltransferase 2A
MeRIP-Seq: Methylated RNA immunoprecipitation sequencing
METTL3/5/14: Methyltransferase 3/5/14
MGAT5: Alpha-1,6-mannosylglycoprotein 6-beta-N-acetylglucosaminyltransferase
MGMT: O-6-Methylguanine-DNA methyltransferase
MKI67: Marker of proliferation Ki-67
MOB3B: MOB kinase activator 3B
MTC: Methyltransferase complex
mTOR: Mechanistic target of rapamycin kinase
MYB: MYB proto-oncogene, transcription factor
MYC: MYC proto-oncogene, BHLH transcription factor
NANOG: Nanog homeobox
NEAT1: Nuclear paraspeckle assembly transcript 1
NPC: NPC intracellular cholesterol transporter 1
OCT4: POU Class 5 Homeobox 1
P53: Tumor protein P53
PCAT6: Prostate cancer associated transcript 6
PD-L1: CD274 molecule
PEG10: Paternally expressed 10
PER1: Period circadian regulator 1
PERP: P53 apoptosis effector related to PMP22
PFKP: Phosphofructokinase, platelet
PI3K: Phosphatidylinositol-4,5-bisphosphate 3-kinase catalytic subunit delta
PKM2: Pyruvate kinase M1/2
PRMT5: Protein arginine methyltransferase 5
PTBP1: Polypyrimidine tract binding protein 1
PTEN: Phosphatase and tensin homolog
PVT1: Pvt1 oncogene
RAB2B: RAB2B, member RAS oncogene family
Raf-1: Raf-1 proto-oncogene
RAGE: Advanced glycosylation end-product specific receptor
RANBP2: RAN binding protein 2
RARA: Retinoic acid receptor alpha
RBM15: RNA binding motif protein 15
RelA: RELA proto-oncogene, NF-KB subunit
RMRP: RNA component of mitochondrial RNA processing endoribonuclease
RNA-seq: RNA sequencing
RUNX1: RUNX family transcription factor 1
SAM: SAM domain, SH3 domain and nuclear localization signals 1
SERPINE2: Serpin family E member 2
SETD7: SET domain containing 7
SLC3A2: Solute carrier family 3 member 2
SLC7A11: Solute carrier family 7 member 11
Snail: Snail family transcriptional repressor 1
snRNA: Small nuclearRNA
SOCS2: Suppressor of cytokine signaling 2
SOX2/4: SRY-box transcription factor 2/4
SPEN: Split-end
SPHK2: Sphingosine kinase 2
SPTBN2: Sphingosine kinase 2
STK4: Serine/threonine kinase 4
TACC3: Transforming acidic coiled-coil containing protein 3
TAF8: TATA-box binding protein associated factor 8
TBL1: Transducin beta like 1 X-linked
TCGA: The cancer genome atlas
TGF-β: Transforming growth factor beta 1
TIMP3: TIMP metallopeptidase inhibitor 3
TK1: Thymidine kinase 1
TMBIM6: Transmembrane BAX inhibitor motif containing 6
TNFR2: TNF receptor superfamily member 1B
TPR: Translocated promoter region, nuclear basket protein
TRAF5: TNF receptor associated factor 5
UBXN1: UBX domain protein 1
UCK2: Uridine-cytidine kinase 2
UHRF2: Ubiquitin like with PHD and ring finger domains 2
USP1/4: Ubiquitin specific peptidase 1/4
VEGFA: Vascular endothelial growth factor A
WIF-1: WNT inhibitory factor 1
WT1: WT1 transcription factor
WTAP: WT1 associated protein
XIST: X inactive specific transcript
YAP: Yes1 associated transcriptional regulator
YPEL5: Yippee like 5
YTHDF1/2/3: YTH N6-methyladenosine RNA binding protein 1/2/3
ZC3H13: Zinc finger CCCH-type containing 13
Zeb1: Zinc finger E-box binding homeobox 1
ZFP217: Zinc finger protein 217
ZMYM1: Zinc finger MYM-type containing 1
