# Supplementary material for: Role of m6A writers, erasers and readers in cancer
Source: Exp Hematol Oncol. 2022 Aug 9;11:45. doi: 10.1186/s40164-022-00298-7 (PMC9361621; doi:10.1186/s40164-022-00298-7)
Supplement: Supplementary file 1 — Additional file 1: Table S1. Role of the modifier in cancer. (Including references). [file 40164_2022_298_MOESM1_ESM.docx]

| **Table S1 Role of the modifier in cancer.** **(Including references)** | | | |
| --- | --- | --- | --- |
| **Type of cancer** | **Role of the modifier** | **m6A(methylation) modifier** | **Reference** |
| Breast Cancer | Oncogene | METTL5, WTAP, VIRMA, ZNF217, FTO, ALKBH5, ELAVL1, YTHDF1/2/3, IGF2BP1/2/3, HNRNPs | [1-38] |
|  | Tumor suppressor | ZC3H13, HAKAI | [39, 40] |
|  | Bivalent | METTL3/14 | [39, 41-49] |
| Lung Cancer | Oncogene | METTL3/5, WTAP, VIRMA, HAKAI, FTO, ELAVL1, YTHDF1, IGF2BP1/3, HNRNPs | [50-84] |
|  | Tumor suppressor | METTL14, YTHDC2 | [85-90] |
|  | Bivalent | ALKBH5, YTHDF2, IGF2BP2 | [72, 85, 91-99] |
| Prostate Cancer | Oncogene | VIRMA, ZNF217, ELAVL1, YTHDF2, IGF2BP2, HNRNPs | [100-111] |
|  | Tumor suppressor | FTO | [112] |
|  | Bivalent | METTL3 | [104, 109, 113-117] |
| Colorectal Cancer | Oncogene | WTAP, ZNF217, HAKAI, ELAVL1, YTHDF1/2/3, YTHDC1/2, IGF2BP1/2/3, HNRNPs, FTO | [118-150] |
|  | Tumor suppressor | METTL14, ZC3H13 | [151-155] |
|  | Bivalent | METTL3, ALKNH5 | [137, 156-166] |
| Gastric Cancer | Oncogene | METTL3/16, WTAP, VIRMA, ZNF217, ALKBH5, ELAVL1, YTHDF1, IGF2BP1/2/3, HNRNPs | [167-199] |
|  | Tumor suppressor | METTL14, YTHDF2 | [200-202] |
|  | Bivalent | FTO | [203-206] |
| Liver Cancer | Oncogene | METTL3, WTAP, VIRMA, ZNF217, FTO, YTHDF1/3, YTHDC2, IGF2BP1/2/3, HNRNPs | [207-250] |
|  | Tumor suppressor | METTL14 | [251-255] |
|  | Bivalent | ALKBH5, ELAVL1, YTHDF2 | [209, 210, 212, 252, 256-262] |
| Cervical Cancer/ Endometrial Cancer/ Ovarian Cancer | Oncogene | ZNF217, WTAP, FTO, ALKBH5, YTHDF1, IGF2BP1/2/3, HNRNPs, ELAVL1, YTHDF2 | [263-310] |
|  | Bivalent | METTL3, YTHDF2 | [289, 311-321] |
| Esophageal Cancer | Oncogene | METTL3, WTAP, FTO, ELAVL1, IGF2BP1/2/3, HNRNPs | [322-338] |
|  | Bivalent | ALKBH5 | [339-341] |
| Thyroid Cancer | Oncogene | METTL14, IGF2BP1/2/3 | [342-346] |
|  | Tumor suppressor | FTO | [347] |
|  | Bivalent | METTL3 | [348, 349] |
| Bladder Cancer | Oncogene | METTL3, WTAP, YTHDF2, IGF2BP1 | [350-355] |
|  | Tumor suppressor | METTL14, ALKBH5 | [356-358] |
|  | Bivalent | FTO | [359, 360] |
| Pancreatic Cancer | Oncogene | METTL3/14, WTAP, YTHDF2, IGF2BP2/3, HNRNPs | [361-377] |
|  | Tumor suppressor | FTO, ALKBH5, YTHDC1 | [368, 378-382] |
| Leukaemia | Oncogene | METTL3/14, WTAP, FTO, ALKBH5, YTHDF2, IGF2BP1/2/3, RBM15 | [383-408] |
| Kidney Cancer | Oncogene | WTAP, IGF2BP1/3, HNRNPs | [409-414] |
|  | Tumor suppressor | METTL14, FTO, YTHDF2 | [415-424] |
|  | Bivalent | ALKBH5 | [422, 425] |
| Melanoma | Oncogene | METTL3, FTO, ALKBH5, ELAVL1, YTHDF1/2, IGF2BP1/2/3, HNRNPs | [426-437] |
| Head and Neck Cancer | Oncogene | METTL3, FTO, ALKBH5, ELAVL1, YTHDF1, IGF2BP1/2//3, HNRNPs | [438-455] |
|  | Bivalent | YTHDC2 | [456, 457] |
| Glioblastoma | Oncogene | METTL3, ZNF217, ALKBH5, ELAVL1, YTHDF1/2, IGF2BP1/2/3 | [458-483] |
| Osteosarcoma | Oncogene | METTL14, ZNF217, ELAVL1, IGF2BP1 | [484-488] |
|  | Tumor suppressor | YTHDF2 | [489] |
|  | Bivalent | ALKBH5 | [490, 491] |
| Cholangiocarcinoma/ Gallbladder Cancer | Oncogene | IGF2BP1/2 | [492, 493] |
|  | Tumor suppressor | FTO, ALKBH5 | [494, 495] |
| Retinoblastoma | Oncogene | METTL3, IGF2BP1 | [496] |
| Lymphomas | Oncogene | METTL3/14, WTAP, RBM15, FTO, ALKBH5, YTHDF2, IGF2BP1/2/3 | [497] |
| Rhabdomyosarcoma | Oncogene | IGF2BP1 | [498] |
| Seminoma | Oncogene | METTL3 | [499] |
| Thymic Epithelial Cancer | Oncogene | METTL3 | [500] |

**References**

1. Rong B, Zhang Q, Wan J, et al. Ribosome 18S m(6)A Methyltransferase METTL5 Promotes Translation Initiation and Breast Cancer Cell Growth. Cell Rep. 2020;33(12):108544.

2. Qian JY, Gao J, Sun X, et al. KIAA1429 acts as an oncogenic factor in breast cancer by regulating CDK1 in an N6-methyladenosine-independent manner. Oncogene. 2019;38(33):6123-6141.

3. Vendrell JA, Thollet A, Nguyen NT, et al. ZNF217 is a marker of poor prognosis in breast cancer that drives epithelial-mesenchymal transition and invasion. Cancer Res. 2012;72(14):3593-3606.

4. Vendrell JA, Solassol J, Gyorffy B, et al. Evaluating ZNF217 mRNA Expression Levels as a Predictor of Response to Endocrine Therapy in ER+ Breast Cancer. Front Pharmacol. 2018;9:1581.

5. Suarez CD, Wu J, Badve SS, et al. The AKT inhibitor triciribine in combination with paclitaxel has order-specific efficacy against Zfp217-induced breast cancer chemoresistance. Oncotarget. 2017;8(65):108534-108547.

6. Northey JJ, Barrett AS, Acerbi I, et al. Stiff stroma increases breast cancer risk by inducing the oncogene ZNF217. J Clin Invest. 2020;130(11):5721-5737.

7. Nonet GH, Stampfer MR, Chin K, et al. The ZNF217 gene amplified in breast cancers promotes immortalization of human mammary epithelial cells. Cancer Res. 2001;61(4):1250-1254.

8. Nguyen NT, Vendrell JA, Poulard C, et al. A functional interplay between ZNF217 and estrogen receptor alpha exists in luminal breast cancers. Mol Oncol. 2014;8(8):1441-1457.

9. Littlepage LE, Adler AS, Kouros-Mehr H, et al. The transcription factor ZNF217 is a prognostic biomarker and therapeutic target during breast cancer progression. Cancer Discov. 2012;2(7):638-651.

10. Frietze S, O'Geen H, Littlepage LE, et al. Global analysis of ZNF217 chromatin occupancy in the breast cancer cell genome reveals an association with ERalpha. BMC Genomics. 2014;15:520.

11. Collins C, Rommens JM, Kowbel D, et al. Positional cloning of ZNF217 and NABC1: genes amplified at 20q13.2 and overexpressed in breast carcinoma. Proc Natl Acad Sci U S A. 1998;95(15):8703-8708.

12. Cohen PA, Loudig O, Liu C, et al. The ZNF217 Biomarker Predicts Low- and High-Risk Oncotype DX((R)) Recurrence Score in ER-Positive Invasive Breast Cancers. Front Pharmacol. 2019;10:524.

13. Bellanger A, Le DT, Vendrell J, et al. Exploring the Significance of the Exon 4-Skipping Isoform of the ZNF217 Oncogene in Breast Cancer. Front Oncol. 2021;11:647269.

14. Bellanger A, Donini CF, Vendrell JA, et al. The critical role of the ZNF217 oncogene in promoting breast cancer metastasis to the bone. J Pathol. 2017;242(1):73-89.

15. Bai WD, Ye XM, Zhang MY, et al. MiR-200c suppresses TGF-beta signaling and counteracts trastuzumab resistance and metastasis by targeting ZNF217 and ZEB1 in breast cancer. Int J Cancer. 2014;135(6):1356-1368.

16. Xu Y, Ye S, Zhang N, et al. The FTO/miR-181b-3p/ARL5B signaling pathway regulates cell migration and invasion in breast cancer. Cancer Commun (Lond). 2020;40(10):484-500.

17. Wang Y, Cheng Z, Xu J, et al. Fat mass and obesity-associated protein (FTO) mediates signal transducer and activator of transcription 3 (STAT3)-drived resistance of breast cancer to doxorubicin. Bioengineered. 2021;12(1):1874-1889.

18. Niu Y, Lin Z, Wan A, et al. RNA N6-methyladenosine demethylase FTO promotes breast tumor progression through inhibiting BNIP3. Mol Cancer. 2019;18(1):46.

19. Zhang C, Zhi WI, Lu H, et al. Hypoxia-inducible factors regulate pluripotency factor expression by ZNF217- and ALKBH5-mediated modulation of RNA methylation in breast cancer cells. Oncotarget. 2016;7(40):64527-64542.

20. Zhang C, Samanta D, Lu H, et al. Hypoxia induces the breast cancer stem cell phenotype by HIF-dependent and ALKBH5-mediated m(6)A-demethylation of NANOG mRNA. Proc Natl Acad Sci U S A. 2016;113(14):E2047-2056.

21. Chou SD, Murshid A, Eguchi T, et al. HSF1 regulation of beta-catenin in mammary cancer cells through control of HuR/elavL1 expression. Oncogene. 2015;34(17):2178-2188.

22. Luo N, Zhang K, Li X, Hu Y. ZEB1 induced-upregulation of long noncoding RNA ZEB1-AS1 facilitates the progression of triple negative breast cancer by binding with ELAVL1 to maintain the stability of ZEB1 mRNA. J Cell Biochem. 2020;121(10):4176-4187.

23. Hu Y, Pan Q, Wang M, et al. m(6)A RNA Methylation Regulator YTHDF1 Correlated With Immune Microenvironment Predicts Clinical Outcomes and Therapeutic Efficacy in Breast Cancer. Front Med (Lausanne). 2021;8:667543.

24. Anita R, Paramasivam A, Priyadharsini JV, Chitra S. The m6A readers YTHDF1 and YTHDF3 aberrations associated with metastasis and predict poor prognosis in breast cancer patients. Am J Cancer Res. 2020;10(8):2546-2554.

25. Einstein JM, Perelis M, Chaim IA, et al. Inhibition of YTHDF2 triggers proteotoxic cell death in MYC-driven breast cancer. Mol Cell. 2021;81(15):3048-3064 e3049.

26. Chang G, Shi L, Ye Y, et al. YTHDF3 Induces the Translation of m(6)A-Enriched Gene Transcripts to Promote Breast Cancer Brain Metastasis. Cancer Cell. 2020;38(6):857-871 e857.

27. Zhu P, He F, Hou Y, et al. A novel hypoxic long noncoding RNA KB-1980E6.3 maintains breast cancer stem cell stemness via interacting with IGF2BP1 to facilitate c-Myc mRNA stability. Oncogene. 2021;40(9):1609-1627.

28. Qiao YS, Zhou JH, Jin BH, et al. LINC00483 is regulated by IGF2BP1 and participates in the progression of breast cancer. Eur Rev Med Pharmacol Sci. 2021;25(3):1379-1386.

29. McMullen ER, Gonzalez ME, Skala SL, et al. CCN6 regulates IGF2BP2 and HMGA2 signaling in metaplastic carcinomas of the breast. Breast Cancer Res Treat. 2018;172(3):577-586.

30. Liu G, Zhu T, Cui Y, et al. Correlation between IGF2BP2 gene polymorphism and the risk of breast cancer in Chinese Han women. Biomed Pharmacother. 2015;69:297-300.

31. Zhang W, Liu H, Jiang J, et al. CircRNA circFOXK2 facilitates oncogenesis in breast cancer via IGF2BP3/miR-370 axis. Aging (Albany NY). 2021;13(14):18978-18992.

32. Wang Z, Tong D, Han C, et al. Blockade of miR-3614 maturation by IGF2BP3 increases TRIM25 expression and promotes breast cancer cell proliferation. EBioMedicine. 2019;41:357-369.

33. Liu Y, Yu C, Wu Y, et al. CD44(+) fibroblasts increases breast cancer cell survival and drug resistance via IGF2BP3-CD44-IGF2 signalling. J Cell Mol Med. 2017;21(9):1979-1988.

34. Bao G, Huang J, Pan W, et al. Long noncoding RNA CERS6-AS1 functions as a malignancy promoter in breast cancer by binding to IGF2BP3 to enhance the stability of CERS6 mRNA. Cancer Med. 2020;9(1):278-289.

35. Petri BJ, Piell KM, South Whitt GC, et al. HNRNPA2B1 regulates tamoxifen- and fulvestrant-sensitivity and hallmarks of endocrine resistance in breast cancer cells. Cancer Lett. 2021;518:152-168.

36. Hu Y, Sun Z, Deng J, et al. Splicing factor hnRNPA2B1 contributes to tumorigenic potential of breast cancer cells through STAT3 and ERK1/2 signaling pathway. Tumour Biol. 2017;39(3):1010428317694318.

37. Gao LB, Zhu XL, Shi JX, et al. HnRNPA2B1 promotes the proliferation of breast cancer MCF-7 cells via the STAT3 pathway. J Cell Biochem. 2021;122(3-4):472-484.

38. Wu Y, Zhao W, Liu Y, et al. Function of HNRNPC in breast cancer cells by controlling the dsRNA-induced interferon response. EMBO J. 2018;37(23).

39. Gong PJ, Shao YC, Yang Y, et al. Analysis of N6-Methyladenosine Methyltransferase Reveals METTL14 and ZC3H13 as Tumor Suppressor Genes in Breast Cancer. Front Oncol. 2020;10:578963.

40. Gong EY, Park E, Lee K. Hakai acts as a coregulator of estrogen receptor alpha in breast cancer cells. Cancer Sci. 2010;101(9):2019-2025.

41. Zhao C, Ling X, Xia Y, et al. The m6A methyltransferase METTL3 controls epithelial-mesenchymal transition, migration and invasion of breast cancer through the MALAT1/miR-26b/HMGA2 axis. Cancer Cell Int. 2021;21(1):441.

42. Xie J, Ba J, Zhang M, et al. The m6A methyltransferase METTL3 promotes the stemness and malignant progression of breast cancer by mediating m6A modification on SOX2. J BUON. 2021;26(2):444-449.

43. Wang H, Xu B, Shi J. N6-methyladenosine METTL3 promotes the breast cancer progression via targeting Bcl-2. Gene. 2020;722:144076.

44. Pan X, Hong X, Li S, et al. METTL3 promotes adriamycin resistance in MCF-7 breast cancer cells by accelerating pri-microRNA-221-3p maturation in a m6A-dependent manner. Exp Mol Med. 2021;53(1):91-102.

45. Cheng L, Zhang X, Huang YZ, et al. Metformin exhibits antiproliferation activity in breast cancer via miR-483-3p/METTL3/m(6)A/p21 pathway. Oncogenesis. 2021;10(1):7.

46. Cai X, Wang X, Cao C, et al. HBXIP-elevated methyltransferase METTL3 promotes the progression of breast cancer via inhibiting tumor suppressor let-7g. Cancer Lett. 2018;415:11-19.

47. Shi Y, Zheng C, Jin Y, et al. Reduced Expression of METTL3 Promotes Metastasis of Triple-Negative Breast Cancer by m6A Methylation-Mediated COL3A1 Up-Regulation. Front Oncol. 2020;10:1126.

48. Yi D, Wang R, Shi X, et al. METTL14 promotes the migration and invasion of breast cancer cells by modulating N6methyladenosine and hsamiR146a5p expression. Oncol Rep. 2020;43(5):1375-1386.

49. Sun T, Wu Z, Wang X, et al. LNC942 promoting METTL14-mediated m(6)A methylation in breast cancer cell proliferation and progression. Oncogene. 2020;39(31):5358-5372.

50. Wu H, Li F, Zhu R. miR-338-5p inhibits cell growth and migration via inhibition of the METTL3/m6A/c-Myc pathway in lung cancer. Acta Biochim Biophys Sin (Shanghai). 2021;53(3):304-316.

51. Wei W, Huo B, Shi X. miR-600 inhibits lung cancer via downregulating the expression of METTL3. Cancer Manag Res. 2019;11:1177-1187.

52. Wanna-Udom S, Terashima M, Lyu H, et al. The m6A methyltransferase METTL3 contributes to Transforming Growth Factor-beta-induced epithelial-mesenchymal transition of lung cancer cells through the regulation of JUNB. Biochem Biophys Res Commun. 2020;524(1):150-155.

53. Cheng C, Wu Y, Xiao T, et al. METTL3-mediated m(6)A modification of ZBTB4 mRNA is involved in the smoking-induced EMT in cancer of the lung. Mol Ther Nucleic Acids. 2021;23:487-500.

54. Chen WW, Qi JW, Hang Y, et al. Simvastatin is beneficial to lung cancer progression by inducing METTL3-induced m6A modification on EZH2 mRNA. Eur Rev Med Pharmacol Sci. 2020;24(8):4263-4270.

55. Xue L, Li J, Lin Y, et al. m(6) A transferase METTL3-induced lncRNA ABHD11-AS1 promotes the Warburg effect of non-small-cell lung cancer. J Cell Physiol. 2021;236(4):2649-2658.

56. Li M, Wang Q, Zhang X, et al. CircPUM1 promotes cell growth and glycolysis in NSCLC via up-regulating METTL3 expression through miR-590-5p. Cell Cycle. 2021;20(13):1279-1294.

57. Yan X, Zhao X, Yan Q, et al. Analysis of the role of METTL5 as a hub gene in lung adenocarcinoma based on a weighted gene co-expression network. Math Biosci Eng. 2021;18(5):6608-6619.

58. Weng L, Qiu K, Gao W, et al. LncRNA PCGEM1 accelerates non-small cell lung cancer progression via sponging miR-433-3p to upregulate WTAP. BMC Pulm Med. 2020;20(1):213.

59. Tang J, Han T, Tong W, et al. N(6)-methyladenosine (m(6)A) methyltransferase KIAA1429 accelerates the gefitinib resistance of non-small-cell lung cancer. Cell Death Discov. 2021;7(1):108.

60. Xu Y, Chen Y, Yao Y, et al. VIRMA contributes to non-small cell lung cancer progression via N(6)-methyladenosine-dependent DAPK3 post-transcriptional modification. Cancer Lett. 2021;522:142-154.

61. Liu Z, Wu Y, Tao Z, Ma L. E3 ubiquitin ligase Hakai regulates cell growth and invasion, and increases the chemosensitivity to cisplatin in nonsmallcell lung cancer cells. Int J Mol Med. 2018;42(2):1145-1151.

62. Wang Y, Li M, Zhang L, et al. m6A demethylase FTO induces NELL2 expression by inhibiting E2F1 m6A modification leading to metastasis of non-small cell lung cancer. Mol Ther Oncolytics. 2021;21:367-376.

63. Shi H, Zhao J, Han L, et al. Retrospective study of gene signatures and prognostic value of m6A regulatory factor in non-small cell lung cancer using TCGA database and the verification of FTO. Aging (Albany NY). 2020;12(17):17022-17037.

64. Mo WL, Deng LJ, Cheng Y, et al. Circular RNA hsa_circ_0072309 promotes tumorigenesis and invasion by regulating the miR-607/FTO axis in non-small cell lung carcinoma. Aging (Albany NY). 2021;13(8):11629-11645.

65. Liu J, Ren D, Du Z, et al. m(6)A demethylase FTO facilitates tumor progression in lung squamous cell carcinoma by regulating MZF1 expression. Biochem Biophys Res Commun. 2018;502(4):456-464.

66. Li J, Han Y, Zhang H, et al. The m6A demethylase FTO promotes the growth of lung cancer cells by regulating the m6A level of USP7 mRNA. Biochem Biophys Res Commun. 2019;512(3):479-485.

67. Ding Y, Qi N, Wang K, et al. FTO Facilitates Lung Adenocarcinoma Cell Progression by Activating Cell Migration Through mRNA Demethylation. Onco Targets Ther. 2020;13:1461-1470.

68. Xie W, Wang Y, Zhang Y, et al. Single-nucleotide polymorphism rs4142441 and MYC co-modulated long non-coding RNA OSER1-AS1 suppresses non-small cell lung cancer by sequestering ELAVL1. Cancer Sci. 2021;112(6):2272-2286.

69. Ni ZZ, He JK, Tang X, et al. Identification of ELAVL1 gene and miRNA-139-3p involved in the aggressiveness of NSCLC. Eur Rev Med Pharmacol Sci. 2020;24(18):9453-9464.

70. Mao G, Mu Z, Wu D. Exosomal lncRNA FOXD3-AS1 upregulates ELAVL1 expression and activates PI3K/Akt pathway to enhance lung cancer cell proliferation, invasion, and 5-fluorouracil resistance. Acta Biochim Biophys Sin (Shanghai). 2021.

71. Zhou J, Xiao D, Qiu T, et al. Loading MicroRNA-376c in Extracellular Vesicles Inhibits Properties of Non-Small Cell Lung Cancer Cells by Targeting YTHDF1. Technol Cancer Res Treat. 2020;19:1533033820977525.

72. Tsuchiya K, Yoshimura K, Inoue Y, et al. YTHDF1 and YTHDF2 are associated with better patient survival and an inflamed tumor-immune microenvironment in non-small-cell lung cancer. Oncoimmunology. 2021;10(1):1962656.

73. Shi Y, Fan S, Wu M, et al. YTHDF1 links hypoxia adaptation and non-small cell lung cancer progression. Nat Commun. 2019;10(1):4892.

74. Lou X, Ning J, Liu W, et al. YTHDF1 Promotes Cyclin B1 Translation through m(6)A Modulation and Contributes to the Poor Prognosis of Lung Adenocarcinoma with KRAS/TP53 Co-Mutation. Cells. 2021;10(7).

75. Zhang J, Luo W, Chi X, et al. IGF2BP1 silencing inhibits proliferation and induces apoptosis of high glucose-induced non-small cell lung cancer cells by regulating Netrin-1. Arch Biochem Biophys. 2020;693:108581.

76. Wang C, Gu Y, Zhang E, et al. A cancer-testis non-coding RNA LIN28B-AS1 activates driver gene LIN28B by interacting with IGF2BP1 in lung adenocarcinoma. Oncogene. 2019;38(10):1611-1624.

77. Huang Q, Guo H, Wang S, et al. A novel circular RNA, circXPO1, promotes lung adenocarcinoma progression by interacting with IGF2BP1. Cell Death Dis. 2020;11(12):1031.

78. Huang H, Wang D, Guo W, et al. Correlated low IGF2BP1 and FOXM1 expression predicts a good prognosis in lung adenocarcinoma. Pathol Res Pract. 2019;215(7):152433.

79. Gong F, Ren P, Zhang Y, et al. MicroRNAs-491-5p suppresses cell proliferation and invasion by inhibiting IGF2BP1 in non-small cell lung cancer. Am J Transl Res. 2016;8(2):485-495.

80. Zhao W, Lu D, Liu L, et al. Insulin-like growth factor 2 mRNA binding protein 3 (IGF2BP3) promotes lung tumorigenesis via attenuating p53 stability. Oncotarget. 2017;8(55):93672-93687.

81. Xueqing H, Jun Z, Yueqiang J, et al. IGF2BP3 May Contributes to Lung Tumorigenesis by Regulating the Alternative Splicing of PKM. Front Bioeng Biotechnol. 2020;8:679.

82. Guo W, Huai Q, Wan H, et al. Prognostic Impact of IGF2BP3 Expression in Patients with Surgically Resected Lung Adenocarcinoma. DNA Cell Biol. 2021;40(2):316-331.

83. Yu PF, Kang AR, Jing LJ, Wang YM. Long non-coding RNA CACNA1G-AS1 promotes cell migration, invasion and epithelial-mesenchymal transition by HNRNPA2B1 in non-small cell lung cancer. Eur Rev Med Pharmacol Sci. 2018;22(4):993-1002.

84. Dowling P, Pollard D, Larkin A, et al. Abnormal levels of heterogeneous nuclear ribonucleoprotein A2B1 (hnRNPA2B1) in tumour tissue and blood samples from patients diagnosed with lung cancer. Mol Biosyst. 2015;11(3):743-752.

85. Mao J, Qiu H, Guo L. LncRNA HCG11 mediated by METTL14 inhibits the growth of lung adenocarcinoma via IGF2BP2/LATS1. Biochem Biophys Res Commun. 2021;580:74-80.

86. Li F, Zhao J, Wang L, et al. METTL14-Mediated miR-30c-1-3p Maturation Represses the Progression of Lung Cancer via Regulation of MARCKSL1 Expression. Mol Biotechnol. 2021.

87. Wang J, Tan L, Jia B, et al. Downregulation of m(6)A Reader YTHDC2 Promotes the Proliferation and Migration of Malignant Lung Cells via CYLD/NF-kappaB Pathway. Int J Biol Sci. 2021;17(10):2633-2651.

88. Sun S, Han Q, Liang M, et al. Downregulation of m(6) A reader YTHDC2 promotes tumor progression and predicts poor prognosis in non-small cell lung cancer. Thorac Cancer. 2020;11(11):3269-3279.

89. Ma L, Chen T, Zhang X, et al. The m(6)A reader YTHDC2 inhibits lung adenocarcinoma tumorigenesis by suppressing SLC7A11-dependent antioxidant function. Redox Biol. 2021;38:101801.

90. Ma L, Zhang X, Yu K, et al. Targeting SLC3A2 subunit of system XC(-) is essential for m(6)A reader YTHDC2 to be an endogenous ferroptosis inducer in lung adenocarcinoma. Free Radic Biol Med. 2021;168:25-43.

91. Zhu Z, Qian Q, Zhao X, et al. N(6)-methyladenosine ALKBH5 promotes non-small cell lung cancer progress by regulating TIMP3 stability. Gene. 2020;731:144348.

92. Zhang D, Ning J, Okon I, et al. Suppression of m6A mRNA modification by DNA hypermethylated ALKBH5 aggravates the oncological behavior of KRAS mutation/LKB1 loss lung cancer. Cell Death Dis. 2021;12(6):518.

93. Yu H, Zhang Z. ALKBH5-mediated m6A demethylation of lncRNA RMRP plays an oncogenic role in lung adenocarcinoma. Mamm Genome. 2021;32(3):195-203.

94. Chao Y, Shang J, Ji W. ALKBH5-m(6)A-FOXM1 signaling axis promotes proliferation and invasion of lung adenocarcinoma cells under intermittent hypoxia. Biochem Biophys Res Commun. 2020;521(2):499-506.

95. Jin D, Guo J, Wu Y, et al. m(6)A demethylase ALKBH5 inhibits tumor growth and metastasis by reducing YTHDFs-mediated YAP expression and inhibiting miR-107/LATS2-mediated YAP activity in NSCLC. Mol Cancer. 2020;19(1):40.

96. Li Y, Sheng H, Ma F, et al. RNA m(6)A reader YTHDF2 facilitates lung adenocarcinoma cell proliferation and metastasis by targeting the AXIN1/Wnt/beta-catenin signaling. Cell Death Dis. 2021;12(5):479.

97. Ma YS, Shi BW, Guo JH, et al. microRNA-320b suppresses HNF4G and IGF2BP2 expression to inhibit angiogenesis and tumor growth of lung cancer. Carcinogenesis. 2021;42(5):762-771.

98. Huang RS, Zheng YL, Li C, et al. MicroRNA-485-5p suppresses growth and metastasis in non-small cell lung cancer cells by targeting IGF2BP2. Life Sci. 2018;199:104-111.

99. Han X, Chen L, Hu Z, et al. Identification of proteins related with pemetrexed resistance by iTRAQ and PRM-based comparative proteomic analysis and exploration of IGF2BP2 and FOLR1 functions in non-small cell lung cancer cells. J Proteomics. 2021;237:104122.

100. Barros-Silva D, Lobo J, Guimaraes-Teixeira C, et al. VIRMA-Dependent N6-Methyladenosine Modifications Regulate the Expression of Long Non-Coding RNAs CCAT1 and CCAT2 in Prostate Cancer. Cancers (Basel). 2020;12(4).

101. Szczyrba J, Nolte E, Hart M, et al. Identification of ZNF217, hnRNP-K, VEGF-A and IPO7 as targets for microRNAs that are downregulated in prostate carcinoma. Int J Cancer. 2013;132(4):775-784.

102. Jiang X, Zhang C, Qi S, et al. Elevated expression of ZNF217 promotes prostate cancer growth by restraining ferroportin-conducted iron egress. Oncotarget. 2016;7(51):84893-84906.

103. Jiang X, Chen Y, Du E, et al. GATA3-driven expression of miR-503 inhibits prostate cancer progression by repressing ZNF217 expression. Cell Signal. 2016;28(9):1216-1224.

104. Li E, Wei B, Wang X, Kang R. METTL3 enhances cell adhesion through stabilizing integrin beta1 mRNA via an m6A-HuR-dependent mechanism in prostatic carcinoma. Am J Cancer Res. 2020;10(3):1012-1025.

105. Melling N, Taskin B, Hube-Magg C, et al. Cytoplasmic accumulation of ELAVL1 is an independent predictor of biochemical recurrence associated with genomic instability in prostate cancer. Prostate. 2016;76(3):259-272.

106. Li J, Xie H, Ying Y, et al. YTHDF2 mediates the mRNA degradation of the tumor suppressors to induce AKT phosphorylation in N6-methyladenosine-dependent way in prostate cancer. Mol Cancer. 2020;19(1):152.

107. Li J, Meng S, Xu M, et al. Downregulation of N(6)-methyladenosine binding YTHDF2 protein mediated by miR-493-3p suppresses prostate cancer by elevating N(6)-methyladenosine levels. Oncotarget. 2018;9(3):3752-3764.

108. Du C, Lv C, Feng Y, Yu S. Activation of the KDM5A/miRNA-495/YTHDF2/m6A-MOB3B axis facilitates prostate cancer progression. J Exp Clin Cancer Res. 2020;39(1):223.

109. Lang C, Yin C, Lin K, et al. m(6) A modification of lncRNA PCAT6 promotes bone metastasis in prostate cancer through IGF2BP2-mediated IGF1R mRNA stabilization. Clin Transl Med. 2021;11(6):e426.

110. Liu B, Jiang HY, Yuan T, et al. Enzalutamide-Induced Upregulation of PCAT6 Promotes Prostate Cancer Neuroendocrine Differentiation by Regulating miR-326/HNRNPA2B1 Axis. Front Oncol. 2021;11:650054.

111. Wang S, Xu G, Chao F, et al. HNRNPC Promotes Proliferation, Metastasis and Predicts Prognosis in Prostate Cancer. Cancer Manag Res. 2021;13:7263-7276.

112. Zhu K, Li Y, Xu Y. The FTO m(6)A demethylase inhibits the invasion and migration of prostate cancer cells by regulating total m(6)A levels. Life Sci. 2021;271:119180.

113. Ma XX, Cao ZG, Zhao SL. m6A methyltransferase METTL3 promotes the progression of prostate cancer via m6A-modified LEF1. Eur Rev Med Pharmacol Sci. 2020;24(7):3565-3571.

114. Ma H, Zhang F, Zhong Q, Hou J. METTL3-mediated m6A modification of KIF3C-mRNA promotes prostate cancer progression and is negatively regulated by miR-320d. Aging (Albany NY). 2021;13(18):22332-22344.

115. Chen Y, Pan C, Wang X, et al. Silencing of METTL3 effectively hinders invasion and metastasis of prostate cancer cells. Theranostics. 2021;11(16):7640-7657.

116. Cai J, Yang F, Zhan H, et al. RNA m(6)A Methyltransferase METTL3 Promotes The Growth Of Prostate Cancer By Regulating Hedgehog Pathway. Onco Targets Ther. 2019;12:9143-9152.

117. Cotter KA, Gallon J, Uebersax N, et al. Mapping of m(6)A and Its Regulatory Targets in Prostate Cancer Reveals a METTL3-Low Induction of Therapy Resistance. Mol Cancer Res. 2021;19(8):1398-1411.

118. Zhang J, Tsoi H, Li X, et al. Carbonic anhydrase IV inhibits colon cancer development by inhibiting the Wnt signalling pathway through targeting the WTAP-WT1-TBL1 axis. Gut. 2016;65(9):1482-1493.

119. Liang H, Lin Z, Ye Y, et al. ARRB2 promotes colorectal cancer growth through triggering WTAP. Acta Biochim Biophys Sin (Shanghai). 2021;53(1):85-93.

120. Zhang ZC, Zheng LQ, Pan LJ, et al. ZNF217 is overexpressed and enhances cell migration and invasion in colorectal carcinoma. Asian Pac J Cancer Prev. 2015;16(6):2459-2463.

121. Rooney PH, Boonsong A, McFadyen MC, et al. The candidate oncogene ZNF217 is frequently amplified in colon cancer. J Pathol. 2004;204(3):282-288.

122. Fang Z, Xiong Y, Zhang C, et al. Coexistence of copy number increases of ZNF217 and CYP24A1 in colorectal cancers in a Chinese population. Oncol Lett. 2010;1(5):925-930.

123. Zhou WJ, Geng ZH, Chi S, et al. Slit-Robo signaling induces malignant transformation through Hakai-mediated E-cadherin degradation during colorectal epithelial cell carcinogenesis. Cell Res. 2011;21(4):609-626.

124. Zhang Z, Gao Q, Wang S. Kinase GSK3beta functions as a suppressor in colorectal carcinoma through the FTO-mediated MZF1/c-Myc axis. J Cell Mol Med. 2021;25(5):2655-2665.

125. Yue C, Chen J, Li Z, et al. microRNA-96 promotes occurrence and progression of colorectal cancer via regulation of the AMPKalpha2-FTO-m6A/MYC axis. J Exp Clin Cancer Res. 2020;39(1):240.

126. Li K, Huang F, Li Y, et al. Stabilization of oncogenic transcripts by the IGF2BP3/ELAVL1 complex promotes tumorigenicity in colorectal cancer. Am J Cancer Res. 2020;10(8):2480-2494.

127. Gu C, Zhang M, Sun W, Dong C. Upregulation of miR-324-5p Inhibits Proliferation and Invasion of Colorectal Cancer Cells by Targeting ELAVL1. Oncol Res. 2019;27(5):515-524.

128. Chen J, Wu Y, Luo X, et al. Circular RNA circRHOBTB3 represses metastasis by regulating the HuR-mediated mRNA stability of PTBP1 in colorectal cancer. Theranostics. 2021;11(15):7507-7526.

129. Nishizawa Y, Konno M, Asai A, et al. Oncogene c-Myc promotes epitranscriptome m(6)A reader YTHDF1 expression in colorectal cancer. Oncotarget. 2018;9(7):7476-7486.

130. Chen P, Liu XQ, Lin X, et al. Targeting YTHDF1 effectively re-sensitizes cisplatin-resistant colon cancer cells by modulating GLS-mediated glutamine metabolism. Mol Ther Oncolytics. 2021;20:228-239.

131. Bai Y, Yang C, Wu R, et al. YTHDF1 Regulates Tumorigenicity and Cancer Stem Cell-Like Activity in Human Colorectal Carcinoma. Front Oncol. 2019;9:332.

132. Zhou D, Tang W, Xu Y, et al. METTL3/YTHDF2 m6A axis accelerates colorectal carcinogenesis through epigenetically suppressing YPEL5. Mol Oncol. 2021;15(8):2172-2184.

133. Ni W, Yao S, Zhou Y, et al. Long noncoding RNA GAS5 inhibits progression of colorectal cancer by interacting with and triggering YAP phosphorylation and degradation and is negatively regulated by the m(6)A reader YTHDF3. Mol Cancer. 2019;18(1):143.

134. Tang S, Liu Q, Xu M. LINC00857 promotes cell proliferation and migration in colorectal cancer by interacting with YTHDC1 and stabilizing SLC7A5. Oncol Lett. 2021;22(2):578.

135. Tanabe A, Tanikawa K, Tsunetomi M, et al. RNA helicase YTHDC2 promotes cancer metastasis via the enhancement of the efficiency by which HIF-1alpha mRNA is translated. Cancer Lett. 2016;376(1):34-42.

136. Zhang XL, Li KJ, Feng JX, et al. Blocking the IGF2BP1-promoted glucose metabolism of colon cancer cells via direct de-stabilizing mRNA of the LDHA enhances anticancer effects. Mol Ther Nucleic Acids. 2021;23:835-846.

137. Li T, Hu PS, Zuo Z, et al. METTL3 facilitates tumor progression via an m(6)A-IGF2BP2-dependent mechanism in colorectal carcinoma. Mol Cancer. 2019;18(1):112.

138. Ye S, Song W, Xu X, et al. IGF2BP2 promotes colorectal cancer cell proliferation and survival through interfering with RAF-1 degradation by miR-195. FEBS Lett. 2016;590(11):1641-1650.

139. Wu XL, Lu RY, Wang LK, et al. Long noncoding RNA HOTAIR silencing inhibits invasion and proliferation of human colon cancer LoVo cells via regulating IGF2BP2. J Cell Biochem. 2018.

140. Wang Y, Lu JH, Wu QN, et al. LncRNA LINRIS stabilizes IGF2BP2 and promotes the aerobic glycolysis in colorectal cancer. Mol Cancer. 2019;18(1):174.

141. Hou P, Meng S, Li M, et al. LINC00460/DHX9/IGF2BP2 complex promotes colorectal cancer proliferation and metastasis by mediating HMGA1 mRNA stability depending on m6A modification. J Exp Clin Cancer Res. 2021;40(1):52.

142. Gao T, Liu X, He B, et al. Long non-coding RNA 91H regulates IGF2 expression by interacting with IGF2BP2 and promotes tumorigenesis in colorectal cancer. Artif Cells Nanomed Biotechnol. 2020;48(1):664-671.

143. Cui J, Tian J, Wang W, et al. IGF2BP2 promotes the progression of colorectal cancer through a YAP-dependent mechanism. Cancer Sci. 2021;112(10):4087-4099.

144. Zhang Y, Liu X, Yu M, et al. Berberine inhibits proliferation and induces G0/G1 phase arrest in colorectal cancer cells by downregulating IGF2BP3. Life Sci. 2020;260:118413.

145. Yang Z, Zhao F, Gu X, et al. Binding of RNA m6A by IGF2BP3 triggers chemoresistance of HCT8 cells via upregulation of ABCB1. Am J Cancer Res. 2021;11(4):1428-1445.

146. Yang Z, Wang T, Wu D, et al. RNA N6-methyladenosine reader IGF2BP3 regulates cell cycle and angiogenesis in colon cancer. J Exp Clin Cancer Res. 2020;39(1):203.

147. Xu W, Sheng Y, Guo Y, et al. Increased IGF2BP3 expression promotes the aggressive phenotypes of colorectal cancer cells in vitro and vivo. J Cell Physiol. 2019;234(10):18466-18479.

148. Lochhead P, Imamura Y, Morikawa T, et al. Insulin-like growth factor 2 messenger RNA binding protein 3 (IGF2BP3) is a marker of unfavourable prognosis in colorectal cancer. Eur J Cancer. 2012;48(18):3405-3413.

149. Huang YY, Zhang CM, Dai YB, et al. USP11 facilitates colorectal cancer proliferation and metastasis by regulating IGF2BP3 stability. Am J Transl Res. 2021;13(2):480-496.

150. Zhang Y, Huang W, Yuan Y, et al. Long non-coding RNA H19 promotes colorectal cancer metastasis via binding to hnRNPA2B1. J Exp Clin Cancer Res. 2020;39(1):141.

151. Yang X, Zhang S, He C, et al. METTL14 suppresses proliferation and metastasis of colorectal cancer by down-regulating oncogenic long non-coding RNA XIST. Mol Cancer. 2020;19(1):46.

152. Chen X, Xu M, Xu X, et al. METTL14 Suppresses CRC Progression via Regulating N6-Methyladenosine-Dependent Primary miR-375 Processing. Mol Ther. 2020;28(2):599-612.

153. Chen X, Xu M, Xu X, et al. METTL14-mediated N6-methyladenosine modification of SOX4 mRNA inhibits tumor metastasis in colorectal cancer. Mol Cancer. 2020;19(1):106.

154. Cai C, Long J, Huang Q, et al. M6A "Writer" Gene METTL14: A Favorable Prognostic Biomarker and Correlated With Immune Infiltrates in Rectal Cancer. Front Oncol. 2021;11:615296.

155. Zhu D, Zhou J, Zhao J, et al. ZC3H13 suppresses colorectal cancer proliferation and invasion via inactivating Ras-ERK signaling. J Cell Physiol. 2019;234(6):8899-8907.

156. Yue M, Liu T, Yan G, et al. LINC01605, regulated by the EP300-SMYD2 complex, potentiates the binding between METTL3 and SPTBN2 in colorectal cancer. Cancer Cell Int. 2021;21(1):504.

157. Yang Z, Quan Y, Chen Y, et al. Knockdown of RNA N6-methyladenosine methyltransferase METTL3 represses Warburg effect in colorectal cancer via regulating HIF-1alpha. Signal Transduct Target Ther. 2021;6(1):89.

158. Xiang S, Liang X, Yin S, et al. N6-methyladenosine methyltransferase METTL3 promotes colorectal cancer cell proliferation through enhancing MYC expression. Am J Transl Res. 2020;12(5):1789-1806.

159. Wen J, Zhang G, Meng Y, et al. RNA m(6)A methyltransferase METTL3 promotes colorectal cancer cell proliferation and invasion by regulating Snail expression. Oncol Lett. 2021;22(4):711.

160. Peng W, Li J, Chen R, et al. Upregulated METTL3 promotes metastasis of colorectal Cancer via miR-1246/SPRED2/MAPK signaling pathway. J Exp Clin Cancer Res. 2019;38(1):393.

161. Lan H, Liu Y, Liu J, et al. Tumor-Associated Macrophages Promote Oxaliplatin Resistance via METTL3-Mediated m(6)A of TRAF5 and Necroptosis in Colorectal Cancer. Mol Pharm. 2021;18(3):1026-1037.

162. Chen H, Gao S, Liu W, et al. RNA N(6)-Methyladenosine Methyltransferase METTL3 Facilitates Colorectal Cancer by Activating the m(6)A-GLUT1-mTORC1 Axis and Is a Therapeutic Target. Gastroenterology. 2021;160(4):1284-1300 e1216.

163. Xu J, Chen Q, Tian K, et al. m6A methyltransferase METTL3 maintains colon cancer tumorigenicity by suppressing SOCS2 to promote cell proliferation. Oncol Rep. 2020;44(3):973-986.

164. Deng R, Cheng Y, Ye S, et al. m(6)A methyltransferase METTL3 suppresses colorectal cancer proliferation and migration through p38/ERK pathways. Onco Targets Ther. 2019;12:4391-4402.

165. Yang P, Wang Q, Liu A, et al. ALKBH5 Holds Prognostic Values and Inhibits the Metastasis of Colon Cancer. Pathol Oncol Res. 2020;26(3):1615-1623.

166. Guo T, Liu DF, Peng SH, Xu AM. ALKBH5 promotes colon cancer progression by decreasing methylation of the lncRNA NEAT1. Am J Transl Res. 2020;12(8):4542-4549.

167. Zhang F, Yan Y, Cao X, et al. Methylation of microRNA-338-5p by EED promotes METTL3-mediated translation of oncogene CDCP1 in gastric cancer. Aging (Albany NY). 2021;13(8):12224-12238.

168. Yue B, Song C, Yang L, et al. METTL3-mediated N6-methyladenosine modification is critical for epithelial-mesenchymal transition and metastasis of gastric cancer. Mol Cancer. 2019;18(1):142.

169. Yang Z, Jiang X, Li D, Jiang X. HBXIP promotes gastric cancer via METTL3-mediated MYC mRNA m6A modification. Aging (Albany NY). 2020;12(24):24967-24982.

170. Yang DD, Chen ZH, Yu K, et al. METTL3 Promotes the Progression of Gastric Cancer via Targeting the MYC Pathway. Front Oncol. 2020;10:115.

171. Wang Q, Chen C, Ding Q, et al. METTL3-mediated m(6)A modification of HDGF mRNA promotes gastric cancer progression and has prognostic significance. Gut. 2020;69(7):1193-1205.

172. Song C, Zhou C. HOXA10 mediates epithelial-mesenchymal transition to promote gastric cancer metastasis partly via modulation of TGFB2/Smad/METTL3 signaling axis. J Exp Clin Cancer Res. 2021;40(1):62.

173. Liu T, Yang S, Sui J, et al. Dysregulated N6-methyladenosine methylation writer METTL3 contributes to the proliferation and migration of gastric cancer. J Cell Physiol. 2020;235(1):548-562.

174. Lin S, Liu J, Jiang W, et al. METTL3 Promotes the Proliferation and Mobility of Gastric Cancer Cells. Open Med (Wars). 2019;14:25-31.

175. Kang J, Huang X, Dong W, et al. MicroRNA-1269b inhibits gastric cancer development through regulating methyltransferase-like 3 (METTL3). Bioengineered. 2021;12(1):1150-1160.

176. Jiang L, Chen T, Xiong L, et al. Knockdown of m6A methyltransferase METTL3 in gastric cancer cells results in suppression of cell proliferation. Oncol Lett. 2020;20(3):2191-2198.

177. Huo FC, Zhu ZM, Zhu WT, et al. METTL3-mediated m(6)A methylation of SPHK2 promotes gastric cancer progression by targeting KLF2. Oncogene. 2021;40(16):2968-2981.

178. Hu H, Kong Q, Huang XX, et al. Longnon-coding RNA BLACAT2 promotes gastric cancer progression via the miR-193b-5p/METTL3 pathway. J Cancer. 2021;12(11):3209-3221.

179. He H, Wu W, Sun Z, Chai L. MiR-4429 prevented gastric cancer progression through targeting METTL3 to inhibit m(6)A-caused stabilization of SEC62. Biochem Biophys Res Commun. 2019;517(4):581-587.

180. Cheng Z, Gao S, Liang X, et al. Inhibiting PP2Acalpha Promotes the Malignant Phenotype of Gastric Cancer Cells through the ATM/METTL3 Axis. Biomed Res Int. 2021;2021:1015293.

181. Wang XK, Zhang YW, Wang CM, et al. METTL16 promotes cell proliferation by up-regulating cyclin D1 expression in gastric cancer. J Cell Mol Med. 2021;25(14):6602-6617.

182. Li H, Su Q, Li B, et al. High expression of WTAP leads to poor prognosis of gastric cancer by influencing tumour-associated T lymphocyte infiltration. J Cell Mol Med. 2020;24(8):4452-4465.

183. Miao R, Dai CC, Mei L, et al. KIAA1429 regulates cell proliferation by targeting c-Jun messenger RNA directly in gastric cancer. J Cell Physiol. 2020;235(10):7420-7432.

184. Yang D, Chang S, Li F, et al. m(6) A transferase KIAA1429-stabilized LINC00958 accelerates gastric cancer aerobic glycolysis through targeting GLUT1. IUBMB LIFE. 2021.

185. Shida A, Fujioka S, Kurihara H, et al. Prognostic significance of ZNF217 expression in gastric carcinoma. Anticancer Res. 2014;34(9):4813-4817.

186. Qiang F, Li J. CircCSNK1G1 Contributes to the Tumorigenesis of Gastric Cancer by Sponging miR-758 and Regulating ZNF217 Expression. Cancer Manag Res. 2021;13:5027-5038.

187. Zhang J, Guo S, Piao HY, et al. ALKBH5 promotes invasion and metastasis of gastric cancer by decreasing methylation of the lncRNA NEAT1. J Physiol Biochem. 2019;75(3):379-389.

188. Pi J, Wang W, Ji M, et al. YTHDF1 Promotes Gastric Carcinogenesis by Controlling Translation of FZD7. Cancer Res. 2021;81(10):2651-2665.

189. Liu T, Yang S, Cheng YP, et al. The N6-Methyladenosine (m6A) Methylation Gene YTHDF1 Reveals a Potential Diagnostic Role for Gastric Cancer. Cancer Manag Res. 2020;12:11953-11964.

190. Chen XY, Liang R, Yi YC, et al. The m(6)A Reader YTHDF1 Facilitates the Tumorigenesis and Metastasis of Gastric Cancer via USP14 Translation in an m(6)A-Dependent Manner. Front Cell Dev Biol. 2021;9:647702.

191. Yang DL, Dong LF, Qiu YB, Luo GY. An oncogenic lncRNA, GLCC1, promotes tumorigenesis in gastric carcinoma by enhancing the c-Myc/IGF2BP1 interaction. Neoplasma. 2021;68(5):1052-1062.

192. Tang W, Chen S, Liu J, et al. Investigation of IGF1, IGF2BP2, and IGFBP3 variants with lymph node status and esophagogastric junction adenocarcinoma risk. J Cell Biochem. 2019;120(4):5510-5518.

193. Shen H, Zhu H, Chen Y, et al. ZEB1-induced LINC01559 expedites cell proliferation, migration and EMT process in gastric cancer through recruiting IGF2BP2 to stabilize ZEB1 expression. Cell Death Dis. 2021;12(4):349.

194. Zhou Y, Huang T, Siu HL, et al. IGF2BP3 functions as a potential oncogene and is a crucial target of miR-34a in gastric carcinogenesis. Mol Cancer. 2017;16(1):77.

195. Zhang J, Ding F, Jiao D, et al. The Aberrant Expression of MicroRNA-125a-5p/IGF2BP3 Axis in Advanced Gastric Cancer and Its Clinical Relevance. Technol Cancer Res Treat. 2020;19:1533033820917332.

196. Jiang L, Li Y, He Y, et al. Knockdown of m6A Reader IGF2BP3 Inhibited Hypoxia-Induced Cell Migration and Angiogenesis by Regulating Hypoxia Inducible Factor-1alpha in Stomach Cancer. Front Oncol. 2021;11:711207.

197. Ishii S, Yamashita K, Harada H, et al. The H19-PEG10/IGF2BP3 axis promotes gastric cancer progression in patients with high lymph node ratios. Oncotarget. 2017;8(43):74567-74581.

198. Peng WZ, Zhao J, Liu X, et al. hnRNPA2B1 regulates the alternative splicing of BIRC5 to promote gastric cancer progression. Cancer Cell Int. 2021;21(1):281.

199. Huang H, Han Y, Zhang C, et al. HNRNPC as a candidate biomarker for chemoresistance in gastric cancer. Tumour Biol. 2016;37(3):3527-3534.

200. Yao Q, He L, Gao X, et al. The m6A Methyltransferase METTL14-Mediated N6-Methyladenosine Modification of PTEN mRNA Inhibits Tumor Growth and Metastasis in Stomach Adenocarcinoma. Front Oncol. 2021;11:699749.

201. Liu X, Xiao M, Zhang L, et al. The m6A methyltransferase METTL14 inhibits the proliferation, migration, and invasion of gastric cancer by regulating the PI3K/AKT/mTOR signaling pathway. J Clin Lab Anal. 2021;35(3):e23655.

202. Shen X, Zhao K, Xu L, et al. YTHDF2 Inhibits Gastric Cancer Cell Growth by Regulating FOXC2 Signaling Pathway. Front Genet. 2020;11:592042.

203. Yang Z, Jiang X, Zhang Z, et al. HDAC3-dependent transcriptional repression of FOXA2 regulates FTO/m6A/MYC signaling to contribute to the development of gastric cancer. Cancer Gene Ther. 2021;28(1-2):141-155.

204. Wang D, Qu X, Lu W, et al. N(6)-Methyladenosine RNA Demethylase FTO Promotes Gastric Cancer Metastasis by Down-Regulating the m6A Methylation of ITGB1. Front Oncol. 2021;11:681280.

205. Feng S, Qiu G, Yang L, et al. Omeprazole improves chemosensitivity of gastric cancer cells by m6A demethylase FTO-mediated activation of mTORC1 and DDIT3 up-regulation. Biosci Rep. 2021;41(1).

206. Li Y, Zheng D, Wang F, et al. Expression of Demethylase Genes, FTO and ALKBH1, Is Associated with Prognosis of Gastric Cancer. Dig Dis Sci. 2019;64(6):1503-1513.

207. Chen H, Duan F, Wang M, et al. Polymorphisms in METTL3 gene and hepatoblastoma risk in Chinese children: A seven-center case-control study. Gene. 2021;800:145834.

208. Wang A, Chen X, Li D, et al. METTL3-mediated m6A methylation of ASPM drives hepatocellular carcinoma cells growth and metastasis. J Clin Lab Anal. 2021;35(9):e23931.

209. Chen M, Wei L, Law CT, et al. RNA N6-methyladenosine methyltransferase-like 3 promotes liver cancer progression through YTHDF2-dependent posttranscriptional silencing of SOCS2. Hepatology. 2018;67(6):2254-2270.

210. Chen Y, Peng C, Chen J, et al. WTAP facilitates progression of hepatocellular carcinoma via m6A-HuR-dependent epigenetic silencing of ETS1. Mol Cancer. 2019;18(1):127.

211. Lan T, Li H, Zhang D, et al. KIAA1429 contributes to liver cancer progression through N6-methyladenosine-dependent post-transcriptional modification of GATA3. Mol Cancer. 2019;18(1):186.

212. Liu H, Lan T, Li H, et al. Circular RNA circDLC1 inhibits MMP1-mediated liver cancer progression via interaction with HuR. Theranostics. 2021;11(3):1396-1411.

213. Si W, Zhao Y, Zhou J, et al. The coordination between ZNF217 and LSD1 contributes to hepatocellular carcinoma progress and is negatively regulated by miR-101. Exp Cell Res. 2019;379(1):1-10.

214. Liu M, Jiang K, Lin G, et al. Ajuba inhibits hepatocellular carcinoma cell growth via targeting of beta-catenin and YAP signaling and is regulated by E3 ligase Hakai through neddylation. J Exp Clin Cancer Res. 2018;37(1):165.

215. Ye Z, Wang S, Chen W, et al. Fat mass and obesity-associated protein promotes the tumorigenesis and development of liver cancer. Oncol Lett. 2020;20(2):1409-1417.

216. Li J, Zhu L, Shi Y, et al. m6A demethylase FTO promotes hepatocellular carcinoma tumorigenesis via mediating PKM2 demethylation. Am J Transl Res. 2019;11(9):6084-6092.

217. Bian X, Shi D, Xing K, et al. AMD1 upregulates hepatocellular carcinoma cells stemness by FTO mediated mRNA demethylation. Clin Transl Med. 2021;11(3):e352.

218. Liu J, Wang D, Zhou J, et al. N6-methyladenosine reader YTHDC2 and eraser FTO may determine hepatocellular carcinoma prognoses after transarterial chemoembolization. Arch Toxicol. 2021;95(5):1621-1629.

219. Luo X, Cao M, Gao F, He X. YTHDF1 promotes hepatocellular carcinoma progression via activating PI3K/AKT/mTOR signaling pathway and inducing epithelial-mesenchymal transition. Exp Hematol Oncol. 2021;10(1):35.

220. Liu X, Qin J, Gao T, et al. YTHDF1 Facilitates the Progression of Hepatocellular Carcinoma by Promoting FZD5 mRNA Translation in an m6A-Dependent Manner. Mol Ther Nucleic Acids. 2020;22:750-765.

221. Zhao X, Chen Y, Mao Q, et al. Overexpression of YTHDF1 is associated with poor prognosis in patients with hepatocellular carcinoma. Cancer Biomark. 2018;21(4):859-868.

222. Li Q, Ni Y, Zhang L, et al. HIF-1alpha-induced expression of m6A reader YTHDF1 drives hypoxia-induced autophagy and malignancy of hepatocellular carcinoma by promoting ATG2A and ATG14 translation. Signal Transduct Target Ther. 2021;6(1):76.

223. Chi F, Cao Y, Chen Y. Analysis and Validation of circRNA-miRNA Network in Regulating m(6)A RNA Methylation Modulators Reveals CircMAP2K4/miR-139-5p/YTHDF1 Axis Involving the Proliferation of Hepatocellular Carcinoma. Front Oncol. 2021;11:560506.

224. Bian S, Ni W, Zhu M, et al. Identification and Validation of the N6-Methyladenosine RNA Methylation Regulator YTHDF1 as a Novel Prognostic Marker and Potential Target for Hepatocellular Carcinoma. Front Mol Biosci. 2020;7:604766.

225. Wang M, Yang Y, Yang J, et al. circ_KIAA1429 accelerates hepatocellular carcinoma advancement through the mechanism of m(6)A-YTHDF3-Zeb1. Life Sci. 2020;257:118082.

226. Guo JC, Liu Z, Yang YJ, et al. KDM5B promotes self-renewal of hepatocellular carcinoma cells through the microRNA-448-mediated YTHDF3/ITGA6 axis. J Cell Mol Med. 2021.

227. Jiang T, Li M, Li Q, et al. MicroRNA-98-5p Inhibits Cell Proliferation and Induces Cell Apoptosis in Hepatocellular Carcinoma via Targeting IGF2BP1. Oncol Res. 2017;25(7):1117-1127.

228. Rebucci M, Sermeus A, Leonard E, et al. miRNA-196b inhibits cell proliferation and induces apoptosis in HepG2 cells by targeting IGF2BP1. Mol Cancer. 2015;14:79.

229. Wei L, Ling M, Yang S, et al. Long noncoding RNA NBAT1 suppresses hepatocellular carcinoma progression via competitively associating with IGF2BP1 and decreasing c-Myc expression. Hum Cell. 2021;34(2):539-549.

230. Xu Y, Zheng Y, Liu H, Li T. Modulation of IGF2BP1 by long non-coding RNA HCG11 suppresses apoptosis of hepatocellular carcinoma cells via MAPK signaling transduction. Int J Oncol. 2017;51(3):791-800.

231. Yang J, Gong X, Yang J, et al. Suppressive role of microRNA-29 in hepatocellular carcinoma via targeting IGF2BP1. Int J Clin Exp Pathol. 2018;11(3):1175-1185.

232. Zhou X, Zhang CZ, Lu SX, et al. miR-625 suppresses tumour migration and invasion by targeting IGF2BP1 in hepatocellular carcinoma. Oncogene. 2015;34(8):965-977.

233. Zhang J, Hu K, Yang YQ, et al. LIN28B-AS1-IGF2BP1 binding promotes hepatocellular carcinoma cell progression. Cell Death Dis. 2020;11(9):741.

234. Zhang J, Cheng J, Zeng Z, et al. Comprehensive profiling of novel microRNA-9 targets and a tumor suppressor role of microRNA-9 via targeting IGF2BP1 in hepatocellular carcinoma. Oncotarget. 2015;6(39):42040-42052.

235. Yang Y, Wu J, Liu F, et al. IGF2BP1 promotes the liver cancer stem cell phenotype by regulating MGAT5 mRNA stability via m6A RNA methylation. Stem Cells Dev. 2021.

236. Yan Y, Huang P, Mao K, et al. Anti-oncogene PTPN13 inactivation by hepatitis B virus X protein counteracts IGF2BP1 to promote hepatocellular carcinoma progression. Oncogene. 2021;40(1):28-45.

237. He J, Zuo Q, Hu B, et al. A novel, liver-specific long noncoding RNA LINC01093 suppresses HCC progression by interaction with IGF2BP1 to facilitate decay of GLI1 mRNA. Cancer Lett. 2019;450:98-109.

238. Gutschner T, Hammerle M, Pazaitis N, et al. Insulin-like growth factor 2 mRNA-binding protein 1 (IGF2BP1) is an important protumorigenic factor in hepatocellular carcinoma. Hepatology. 2014;59(5):1900-1911.

239. Fawzy IO, Hamza MT, Hosny KA, et al. Abrogating the interplay between IGF2BP1, 2 and 3 and IGF1R by let-7i arrests hepatocellular carcinoma growth. Growth Factors. 2016;34(1-2):42-50.

240. Wei Q. Bioinformatical identification of key genes regulated by IGF2BP2-mediated RNA N6-methyladenosine and prediction of prognosis in hepatocellular carcinoma. J Gastrointest Oncol. 2021;12(4):1773-1785.

241. Pu J, Wang J, Qin Z, et al. IGF2BP2 Promotes Liver Cancer Growth Through an m6A-FEN1-Dependent Mechanism. Front Oncol. 2020;10:578816.

242. Liu FY, Zhou SJ, Deng YL, et al. MiR-216b is involved in pathogenesis and progression of hepatocellular carcinoma through HBx-miR-216b-IGF2BP2 signaling pathway. Cell Death Dis. 2015;6:e1670.

243. Fen H, Hongmin Z, Wei W, et al. RHPN1-AS1 Drives the Progression of Hepatocellular Carcinoma via Regulating miR-596/IGF2BP2 Axis. Curr Pharm Des. 2020;25(43):4630-4640.

244. Zhang W, Zhu L, Yang G, et al. Hsa_circ_0026134 expression promoted TRIM25- and IGF2BP3-mediated hepatocellular carcinoma cell proliferation and invasion via sponging miR-127-5p. Biosci Rep. 2020;40(7).

245. Li M, Zhang L, Ge C, et al. An isocorydine derivative (d-ICD) inhibits drug resistance by downregulating IGF2BP3 expression in hepatocellular carcinoma. Oncotarget. 2015;6(28):25149-25160.

246. Jiang W, Cheng X, Wang T, et al. LINC00467 promotes cell proliferation and metastasis by binding with IGF2BP3 to enhance the mRNA stability of TRAF5 in hepatocellular carcinoma. J Gene Med. 2020;22(3):e3134.

247. Gao Y, Luo T, Ouyang X, et al. IGF2BP3 and miR191-5p synergistically increase HCC cell invasiveness by altering ZO-1 expression. Oncol Lett. 2020;20(2):1423-1431.

248. Wang H, Liang L, Dong Q, et al. Long noncoding RNA miR503HG, a prognostic indicator, inhibits tumor metastasis by regulating the HNRNPA2B1/NF-kappaB pathway in hepatocellular carcinoma. Theranostics. 2018;8(10):2814-2829.

249. Luo J, Zheng J, Hao W, et al. lncRNA PCAT6 facilitates cell proliferation and invasion via regulating the miR-326/hnRNPA2B1 axis in liver cancer. Oncol Lett. 2021;21(6):471.

250. Chen T, Gu C, Xue C, et al. LncRNA-uc002mbe.2 Interacting with hnRNPA2B1 Mediates AKT Deactivation and p21 Up-Regulation Induced by Trichostatin in Liver Cancer Cells. Front Pharmacol. 2017;8:669.

251. Du L, Li Y, Kang M, et al. USP48 Is Upregulated by Mettl14 to Attenuate Hepatocellular Carcinoma via Regulating SIRT6 Stabilization. Cancer Res. 2021;81(14):3822-3834.

252. Fan Z, Yang G, Zhang W, et al. Hypoxia blocks ferroptosis of hepatocellular carcinoma via suppression of METTL14 triggered YTHDF2-dependent silencing of SLC7A11. J Cell Mol Med. 2021.

253. Liu X, Qin J, Gao T, et al. Analysis of METTL3 and METTL14 in hepatocellular carcinoma. Aging (Albany NY). 2020;12(21):21638-21659.

254. Ma JZ, Yang F, Zhou CC, et al. METTL14 suppresses the metastatic potential of hepatocellular carcinoma by modulating N(6) -methyladenosine-dependent primary MicroRNA processing. Hepatology. 2017;65(2):529-543.

255. Shi Y, Zhuang Y, Zhang J, et al. METTL14 Inhibits Hepatocellular Carcinoma Metastasis Through Regulating EGFR/PI3K/AKT Signaling Pathway in an m6A-Dependent Manner. Cancer Manag Res. 2020;12:13173-13184.

256. Qu S, Jin L, Huang H, et al. A positive-feedback loop between HBx and ALKBH5 promotes hepatocellular carcinogenesis. BMC Cancer. 2021;21(1):686.

257. Liu Z, Wang Q, Wang X, et al. Circular RNA cIARS regulates ferroptosis in HCC cells through interacting with RNA binding protein ALKBH5. Cell Death Discov. 2020;6:72.

258. Chen Y, Zhao Y, Chen J, et al. ALKBH5 suppresses malignancy of hepatocellular carcinoma via m(6)A-guided epigenetic inhibition of LYPD1. Mol Cancer. 2020;19(1):123.

259. Shi J, Guo C, Ma J. CCAT2 enhances autophagy-related invasion and metastasis via regulating miR-4496 and ELAVL1 in hepatocellular carcinoma. J Cell Mol Med. 2021;25(18):8985-8996.

260. Zhong L, Liao D, Zhang M, et al. YTHDF2 suppresses cell proliferation and growth via destabilizing the EGFR mRNA in hepatocellular carcinoma. Cancer Lett. 2019;442:252-261.

261. Hou J, Zhang H, Liu J, et al. YTHDF2 reduction fuels inflammation and vascular abnormalization in hepatocellular carcinoma. Mol Cancer. 2019;18(1):163.

262. Zhang C, Huang S, Zhuang H, et al. YTHDF2 promotes the liver cancer stem cell phenotype and cancer metastasis by regulating OCT4 expression via m6A RNA methylation. Oncogene. 2020;39(23):4507-4518.

263. Yu HL, Ma XD, Tong JF, et al. WTAP is a prognostic marker of high-grade serous ovarian cancer and regulates the progression of ovarian cancer cells. Onco Targets Ther. 2019;12:6191-6201.

264. Wang J, Xu J, Li K, et al. Identification of WTAP-related genes by weighted gene co-expression network analysis in ovarian cancer. J Ovarian Res. 2020;13(1):119.

265. Fu Y, Jia XC. WTAP-mediated N6-methyladenosine modification on EGR3 in different types of epithelial ovarian cancer. J Biol Regul Homeost Agents. 2020;34(4):1505-1512.

266. Yu H, Zhao K, Zeng H, et al. N(6)-methyladenosine (m(6)A) methyltransferase WTAP accelerates the Warburg effect of gastric cancer through regulating HK2 stability. Biomed Pharmacother. 2021;133:111075.

267. Li Q, Wang C, Dong W, et al. WTAP facilitates progression of endometrial cancer via CAV-1/NF-kappaB axis. Cell Biol Int. 2021;45(6):1269-1277.

268. Sun Y, Wong N, Guan Y, et al. The eukaryotic translation elongation factor eEF1A2 induces neoplastic properties and mediates tumorigenic effects of ZNF217 in precursor cells of human ovarian carcinomas. Int J Cancer. 2008;123(8):1761-1769.

269. Sun G, Zhou J, Yin A, et al. Silencing of ZNF217 gene influences the biological behavior of a human ovarian cancer cell line. Int J Oncol. 2008;32(5):1065-1071.

270. Sun G, Qin J, Qiu Y, et al. Microarray analysis of gene expression in the ovarian cancer cell line HO-8910 with silencing of the ZNF217 gene. Mol Med Rep. 2009;2(5):851-855.

271. Rahman MT, Nakayama K, Rahman M, et al. Prognostic and therapeutic impact of the chromosome 20q13.2 ZNF217 locus amplification in ovarian clear cell carcinoma. Cancer. 2012;118(11):2846-2857.

272. Rahman MT, Nakayama K, Rahman M, et al. Gene amplification of ZNF217 located at chr20q13.2 is associated with lymph node metastasis in ovarian clear cell carcinoma. Anticancer Res. 2012;32(8):3091-3095.

273. Li P, Maines-Bandiera S, Kuo WL, et al. Multiple roles of the candidate oncogene ZNF217 in ovarian epithelial neoplastic progression. Int J Cancer. 2007;120(9):1863-1873.

274. Li J, Song L, Qiu Y, et al. ZNF217 is associated with poor prognosis and enhances proliferation and metastasis in ovarian cancer. Int J Clin Exp Pathol. 2014;7(6):3038-3047.

275. Krig SR, Miller JK, Frietze S, et al. ZNF217, a candidate breast cancer oncogene amplified at 20q13, regulates expression of the ErbB3 receptor tyrosine kinase in breast cancer cells. Oncogene. 2010;29(40):5500-5510.

276. Huang HN, Lin MC, Huang WC, et al. Loss of ARID1A expression and its relationship with PI3K-Akt pathway alterations and ZNF217 amplification in ovarian clear cell carcinoma. Mod Pathol. 2014;27(7):983-990.

277. Huang HN, Huang WC, Lin CH, et al. Chromosome 20q13.2 ZNF217 locus amplification correlates with decreased E-cadherin expression in ovarian clear cell carcinoma with PI3K-Akt pathway alterations. Hum Pathol. 2014;45(11):2318-2325.

278. Guo L, Chen J, Liu D, Liu L. OIP5-AS1/miR-137/ZNF217 Axis Promotes Malignant Behaviors in Epithelial Ovarian Cancer. Cancer Manag Res. 2020;12:6707-6717.

279. Yang S, Shi F, Du Y, et al. Long non-coding RNA CTBP1-AS2 enhances cervical cancer progression via up-regulation of ZNF217 through sponging miR-3163. Cancer Cell Int. 2020;20:343.

280. Zou D, Dong L, Li C, et al. The m(6)A eraser FTO facilitates proliferation and migration of human cervical cancer cells. Cancer Cell Int. 2019;19:321.

281. Zhou S, Bai ZL, Xia D, et al. FTO regulates the chemo-radiotherapy resistance of cervical squamous cell carcinoma (CSCC) by targeting beta-catenin through mRNA demethylation. Mol Carcinog. 2018;57(5):590-597.

282. Wang T, Li W, Ye B, et al. FTO-stabilized lncRNA HOXC13-AS epigenetically upregulated FZD6 and activated Wnt/beta-catenin signaling to drive cervical cancer proliferation, invasion, and EMT. J BUON. 2021;26(4):1279-1291.

283. Zhang L, Wan Y, Zhang Z, et al. FTO demethylates m6A modifications in HOXB13 mRNA and promotes endometrial cancer metastasis by activating the WNT signalling pathway. RNA Biol. 2021;18(9):1265-1278.

284. Zhu H, Gan X, Jiang X, et al. ALKBH5 inhibited autophagy of epithelial ovarian cancer through miR-7 and BCL-2. J Exp Clin Cancer Res. 2019;38(1):163.

285. Nie S, Zhang L, Liu J, et al. ALKBH5-HOXA10 loop-mediated JAK2 m6A demethylation and cisplatin resistance in epithelial ovarian cancer. J Exp Clin Cancer Res. 2021;40(1):284.

286. Jiang Y, Wan Y, Gong M, et al. RNA demethylase ALKBH5 promotes ovarian carcinogenesis in a simulated tumour microenvironment through stimulating NF-kappaB pathway. J Cell Mol Med. 2020;24(11):6137-6148.

287. Pu X, Gu Z, Gu Z. ALKBH5 regulates IGF1R expression to promote the Proliferation and Tumorigenicity of Endometrial Cancer. J Cancer. 2020;11(19):5612-5622.

288. Xue F, Li QR, Xu YH, Zhou HB. MicroRNA-139-3p Inhibits The Growth And Metastasis Of Ovarian Cancer By Inhibiting ELAVL1. Onco Targets Ther. 2019;12:8935-8945.

289. Wang Q, Guo X, Li L, et al. N(6)-methyladenosine METTL3 promotes cervical cancer tumorigenesis and Warburg effect through YTHDF1/HK2 modification. Cell Death Dis. 2020;11(10):911.

290. Wang H, Luo Q, Kang J, et al. YTHDF1 Aggravates the Progression of Cervical Cancer Through m(6)A-Mediated Up-Regulation of RANBP2. Front Oncol. 2021;11:650383.

291. Xu F, Li J, Ni M, et al. FBW7 suppresses ovarian cancer development by targeting the N(6)-methyladenosine binding protein YTHDF2. Mol Cancer. 2021;20(1):45.

292. Li J, Wu L, Pei M, Zhang Y. YTHDF2, a protein repressed by miR-145, regulates proliferation, apoptosis, and migration in ovarian cancer cells. J Ovarian Res. 2020;13(1):111.

293. Liu T, Wei Q, Jin J, et al. The m6A reader YTHDF1 promotes ovarian cancer progression via augmenting EIF3C translation. Nucleic Acids Res. 2020;48(7):3816-3831.

294. Hao L, Wang JM, Liu BQ, et al. m6A-YTHDF1-mediated TRIM29 upregulation facilitates the stem cell-like phenotype of cisplatin-resistant ovarian cancer cells. Biochim Biophys Acta Mol Cell Res. 2021;1868(1):118878.

295. Zhang L, Wan Y, Zhang Z, et al. IGF2BP1 overexpression stabilizes PEG10 mRNA in an m6A-dependent manner and promotes endometrial cancer progression. Theranostics. 2021;11(3):1100-1114.

296. Xue T, Liu X, Zhang M, et al. PADI2-Catalyzed MEK1 Citrullination Activates ERK1/2 and Promotes IGF2BP1-Mediated SOX2 mRNA Stability in Endometrial Cancer. Adv Sci (Weinh). 2021;8(6):2002831.

297. Qin X, Sun L, Wang J. Restoration of microRNA-708 sensitizes ovarian cancer cells to cisplatin via IGF2BP1/Akt pathway. Cell Biol Int. 2017;41(10):1110-1118.

298. Bley N, Schott A, Muller S, et al. IGF2BP1 is a targetable SRC/MAPK-dependent driver of invasive growth in ovarian cancer. RNA Biol. 2021;18(3):391-403.

299. Wang P, Zhang L, Zhang J, Xu G. MicroRNA-124-3p inhibits cell growth and metastasis in cervical cancer by targeting IGF2BP1. Exp Ther Med. 2018;15(2):1385-1393.

300. Su Y, Xiong J, Hu J, et al. MicroRNA-140-5p targets insulin like growth factor 2 mRNA binding protein 1 (IGF2BP1) to suppress cervical cancer growth and metastasis. Oncotarget. 2016;7(42):68397-68411.

301. Shu J, Wang D. Functional characterization of the long noncoding RNA MIR22HG as a tumour suppressor in cervical cancer by targeting IGF2BP2. Eur Rev Med Pharmacol Sci. 2020;24(15):7953-7962.

302. Ji F, Lu Y, Chen S, et al. IGF2BP2-modified circular RNA circARHGAP12 promotes cervical cancer progression by interacting m(6)A/FOXM1 manner. Cell Death Discov. 2021;7(1):215.

303. Liu H, Zeng Z, Afsharpad M, et al. Overexpression of IGF2BP3 as a Potential Oncogene in Ovarian Clear Cell Carcinoma. Front Oncol. 2019;9:1570.

304. Kobel M, Xu H, Bourne PA, et al. IGF2BP3 (IMP3) expression is a marker of unfavorable prognosis in ovarian carcinoma of clear cell subtype. Mod Pathol. 2009;22(3):469-475.

305. Hsu KF, Shen MR, Huang YF, et al. Overexpression of the RNA-binding proteins Lin28B and IGF2BP3 (IMP3) is associated with chemoresistance and poor disease outcome in ovarian cancer. Br J Cancer. 2015;113(3):414-424.

306. Fadare O, Liang SX, Crispens MA, et al. Expression of the oncofetal protein IGF2BP3 in endometrial clear cell carcinoma: assessment of frequency and significance. Hum Pathol. 2013;44(8):1508-1515.

307. Zhu J, Han S. Downregulation of LncRNA DARS-AS1 Inhibits the Tumorigenesis of Cervical Cancer via Inhibition of IGF2BP3. Onco Targets Ther. 2021;14:1331-1340.

308. Yang Y, Wei Q, Tang Y, et al. Loss of hnRNPA2B1 inhibits malignant capability and promotes apoptosis via down-regulating Lin28B expression in ovarian cancer. Cancer Lett. 2020;475:43-52.

309. Wang JM, Liu BQ, Zhang Q, et al. ISG15 suppresses translation of ABCC2 via ISGylation of hnRNPA2B1 and enhances drug sensitivity in cisplatin resistant ovarian cancer cells. Biochim Biophys Acta Mol Cell Res. 2020;1867(4):118647.

310. Kleemann M, Schneider H, Unger K, et al. MiR-744-5p inducing cell death by directly targeting HNRNPC and NFIX in ovarian cancer cells. Sci Rep. 2018;8(1):9020.

311. Ni HH, Zhang L, Huang H, et al. Connecting METTL3 and intratumoural CD33(+) MDSCs in predicting clinical outcome in cervical cancer. J Transl Med. 2020;18(1):393.

312. Ji F, Lu Y, Chen S, et al. m(6)A methyltransferase METTL3-mediated lncRNA FOXD2-AS1 promotes the tumorigenesis of cervical cancer. Mol Ther Oncolytics. 2021;22:574-581.

313. Hu Y, Li Y, Huang Y, et al. METTL3 regulates the malignancy of cervical cancer via post-transcriptional regulation of RAB2B. Eur J Pharmacol. 2020;879:173134.

314. Wu F, Zhang Y, Fang Y, et al. Elevated Expression of Inhibitor of Apoptosis-stimulating Protein of p53 (iASPP) and Methyltransferase-like 3 (METTL3) Correlate with Poor Prognosis in FIGO Ib1-IIa Squamous Cell Cervical Cancer. J Cancer. 2020;11(9):2382-2389.

315. Li R, Song Y, Chen X, et al. METTL3 increases cisplatin chemosensitivity of cervical cancer cells via downregulation of the activity of RAGE. Mol Ther Oncolytics. 2021;22:245-255.

316. Ma Z, Li Q, Liu P, et al. METTL3 regulates m6A in endometrioid epithelial ovarian cancer independently of METTl14 and WTAP. Cell Biol Int. 2020;44(12):2524-2531.

317. Liang S, Guan H, Lin X, et al. METTL3 serves an oncogenic role in human ovarian cancer cells partially via the AKT signaling pathway. Oncol Lett. 2020;19(4):3197-3204.

318. Bi X, Lv X, Liu D, et al. METTL3-mediated maturation of miR-126-5p promotes ovarian cancer progression via PTEN-mediated PI3K/Akt/mTOR pathway. Cancer Gene Ther. 2021;28(3-4):335-349.

319. Bi X, Lv X, Liu D, et al. METTL3 promotes the initiation and metastasis of ovarian cancer by inhibiting CCNG2 expression via promoting the maturation of pri-microRNA-1246. Cell Death Discov. 2021;7(1):237.

320. Shen J, Feng XP, Hu RB, et al. N-methyladenosine reader YTHDF2-mediated long noncoding RNA FENDRR degradation promotes cell proliferation in endometrioid endometrial carcinoma. Lab Invest. 2021;101(6):775-784.

321. Hong L, Pu X, Gan H, et al. YTHDF2 inhibit the tumorigenicity of endometrial cancer via downregulating the expression of IRS1 methylated with m(6)A. J Cancer. 2021;12(13):3809-3818.

322. Zou J, Zhong X, Zhou X, et al. The M6A methyltransferase METTL3 regulates proliferation in esophageal squamous cell carcinoma. Biochem Biophys Res Commun. 2021;580:48-55.

323. Zhang M, Bai M, Wang L, et al. Targeting SNHG3/miR-186-5p reverses the increased m6A level caused by platinum treatment through regulating METTL3 in esophageal cancer. Cancer Cell Int. 2021;21(1):114.

324. Hou H, Zhao H, Yu X, et al. METTL3 promotes the proliferation and invasion of esophageal cancer cells partly through AKT signaling pathway. Pathol Res Pract. 2020;216(9):153087.

325. Han H, Yang C, Zhang S, et al. METTL3-mediated m(6)A mRNA modification promotes esophageal cancer initiation and progression via Notch signaling pathway. Mol Ther Nucleic Acids. 2021;26:333-346.

326. Chen X, Huang L, Yang T, et al. METTL3 Promotes Esophageal Squamous Cell Carcinoma Metastasis Through Enhancing GLS2 Expression. Front Oncol. 2021;11:667451.

327. Wang W, Shao F, Yang X, et al. METTL3 promotes tumour development by decreasing APC expression mediated by APC mRNA N(6)-methyladenosine-dependent YTHDF binding. Nat Commun. 2021;12(1):3803.

328. Zhu ZJ, Pang Y, Jin G, et al. Hypoxia induces chemoresistance of esophageal cancer cells to cisplatin through regulating the lncRNA-EMS/miR-758-3p/WTAP axis. Aging (Albany NY). 2021;13(13):17155-17176.

329. Cui Y, Zhang C, Ma S, et al. RNA m6A demethylase FTO-mediated epigenetic up-regulation of LINC00022 promotes tumorigenesis in esophageal squamous cell carcinoma. J Exp Clin Cancer Res. 2021;40(1):294.

330. Li H, Zhang C, Zhang M, et al. Angustoline Inhibited Esophageal Tumors Through Regulating LKB1/AMPK/ELAVL1/LPACT2 Pathway and Phospholipid Remodeling. Front Oncol. 2020;10:1094.

331. Fang XY, Sun JJ, Chen SY, et al. IGF2BP1/UHRF2 Axis Mediated by miR-98-5p to Promote the Proliferation of and Inhibit the Apoptosis of Esophageal Squamous Cell Carcinoma. Ann Clin Lab Sci. 2021;51(3):329-338.

332. Wu X, Fan Y, Liu Y, et al. Long Non-Coding RNA CCAT2 Promotes the Development of Esophageal Squamous Cell Carcinoma by Inhibiting miR-200b to Upregulate the IGF2BP2/TK1 Axis. Front Oncol. 2021;11:680642.

333. Huang GW, Chen QQ, Ma CC, et al. linc01305 promotes metastasis and proliferation of esophageal squamous cell carcinoma through interacting with IGF2BP2 and IGF2BP3 to stabilize HTR3A mRNA. Int J Biochem Cell Biol. 2021;136:106015.

334. Barghash A, Golob-Schwarzl N, Helms V, et al. Elevated expression of the IGF2 mRNA binding protein 2 (IGF2BP2/IMP2) is linked to short survival and metastasis in esophageal adenocarcinoma. Oncotarget. 2016;7(31):49743-49750.

335. Wakita A, Motoyama S, Sato Y, et al. IGF2BP3 Expression Correlates With Poor Prognosis in Esophageal Squamous Cell Carcinoma. J Surg Res. 2021;259:137-144.

336. Li K, Chen J, Lou X, et al. HNRNPA2B1 Affects the Prognosis of Esophageal Cancer by Regulating the miR-17-92 Cluster. Front Cell Dev Biol. 2021;9:658642.

337. Guo H, Wang B, Xu K, et al. m(6)A Reader HNRNPA2B1 Promotes Esophageal Cancer Progression via Up-Regulation of ACLY and ACC1. Front Oncol. 2020;10:553045.

338. Zhang Y, Chen W, Pan T, et al. LBX2-AS1 is activated by ZEB1 and promotes the development of esophageal squamous cell carcinoma by interacting with HNRNPC to enhance the stability of ZEB1 and ZEB2 mRNAs. Biochem Biophys Res Commun. 2019;511(3):566-572.

339. Xiao D, Fang TX, Lei Y, et al. m(6)A demethylase ALKBH5 suppression contributes to esophageal squamous cell carcinoma progression. Aging (Albany NY). 2021;13(17):21497-21512.

340. Chen P, Li S, Zhang K, et al. N(6)-methyladenosine demethylase ALKBH5 suppresses malignancy of esophageal cancer by regulating microRNA biogenesis and RAI1 expression. Oncogene. 2021;40(37):5600-5612.

341. Nagaki Y, Motoyama S, Yamaguchi T, et al. m(6) A demethylase ALKBH5 promotes proliferation of esophageal squamous cell carcinoma associated with poor prognosis. Genes Cells. 2020;25(8):547-561.

342. Zhang X, Li D, Jia C, et al. METTL14 promotes tumorigenesis by regulating lncRNA OIP5-AS1/miR-98/ADAMTS8 signaling in papillary thyroid cancer. Cell Death Dis. 2021;12(6):617.

343. Haase J, Misiak D, Bauer M, et al. IGF2BP1 is the first positive marker for anaplastic thyroid carcinoma diagnosis. Mod Pathol. 2021;34(1):32-41.

344. Ye M, Dong S, Hou H, et al. Oncogenic Role of Long Noncoding RNAMALAT1 in Thyroid Cancer Progression through Regulation of the miR-204/IGF2BP2/m6A-MYC Signaling. Mol Ther Nucleic Acids. 2021;23:1-12.

345. Dong L, Geng Z, Liu Z, et al. IGF2BP2 knockdown suppresses thyroid cancer progression by reducing the expression of long non-coding RNA HAGLR. Pathol Res Pract. 2021;225:153550.

346. Panebianco F, Kelly LM, Liu P, et al. THADA fusion is a mechanism of IGF2BP3 activation and IGF1R signaling in thyroid cancer. Proc Natl Acad Sci U S A. 2017;114(9):2307-2312.

347. Tian R, Zhang S, Sun D, et al. M6A Demethylase FTO Plays a Tumor Suppressor Role in Thyroid Cancer. DNA Cell Biol. 2020.

348. Lin S, Zhu Y, Ji C, et al. METTL3-Induced miR-222-3p Upregulation Inhibits STK4 and Promotes the Malignant Behaviors of Thyroid Carcinoma Cells. J Clin Endocrinol Metab. 2021.

349. He J, Zhou M, Yin J, et al. METTL3 restrains papillary thyroid cancer progression via m(6)A/c-Rel/IL-8-mediated neutrophil infiltration. Mol Ther. 2021;29(5):1821-1837.

350. Xie H, Li J, Ying Y, et al. METTL3/YTHDF2 m(6) A axis promotes tumorigenesis by degrading SETD7 and KLF4 mRNAs in bladder cancer. J Cell Mol Med. 2020;24(7):4092-4104.

351. Wang G, Dai Y, Li K, et al. Deficiency of Mettl3 in Bladder Cancer Stem Cells Inhibits Bladder Cancer Progression and Angiogenesis. Front Cell Dev Biol. 2021;9:627706.

352. Han J, Wang JZ, Yang X, et al. METTL3 promote tumor proliferation of bladder cancer by accelerating pri-miR221/222 maturation in m6A-dependent manner. Mol Cancer. 2019;18(1):110.

353. Cheng M, Sheng L, Gao Q, et al. The m(6)A methyltransferase METTL3 promotes bladder cancer progression via AFF4/NF-kappaB/MYC signaling network. Oncogene. 2019;38(19):3667-3680.

354. Chen L, Wang X. Relationship between the genetic expression of WTAP and bladder cancer and patient prognosis. Oncol Lett. 2018;16(6):6966-6970.

355. Xie F, Huang C, Liu F, et al. CircPTPRA blocks the recognition of RNA N(6)-methyladenosine through interacting with IGF2BP1 to suppress bladder cancer progression. Mol Cancer. 2021;20(1):68.

356. Zhang N, Hua X, Tu H, et al. Isorhapontigenin (ISO) inhibits EMT through FOXO3A/METTL14/VIMENTIN pathway in bladder cancer cells. Cancer Lett. 2021;520:400-408.

357. Gu C, Wang Z, Zhou N, et al. Mettl14 inhibits bladder TIC self-renewal and bladder tumorigenesis through N(6)-methyladenosine of Notch1. Mol Cancer. 2019;18(1):168.

358. Yu H, Yang X, Tang J, et al. ALKBH5 Inhibited Cell Proliferation and Sensitized Bladder Cancer Cells to Cisplatin by m6A-CK2alpha-Mediated Glycolysis. Mol Ther Nucleic Acids. 2021;23:27-41.

359. Yi W, Yu Y, Li Y, et al. The tumor-suppressive effects of alpha-ketoglutarate-dependent dioxygenase FTO via N6-methyladenosine RNA methylation on bladder cancer patients. Bioengineered. 2021;12(1):5323-5333.

360. Tao L, Mu X, Chen H, et al. FTO modifies the m6A level of MALAT and promotes bladder cancer progression. Clin Transl Med. 2021;11(2):e310.

361. Taketo K, Konno M, Asai A, et al. The epitranscriptome m6A writer METTL3 promotes chemo- and radioresistance in pancreatic cancer cells. Int J Oncol. 2018;52(2):621-629.

362. Xia T, Wu X, Cao M, et al. The RNA m6A methyltransferase METTL3 promotes pancreatic cancer cell proliferation and invasion. Pathol Res Pract. 2019;215(11):152666.

363. Zhang C, Ou S, Zhou Y, et al. m(6)A Methyltransferase METTL14-Mediated Upregulation of Cytidine Deaminase Promoting Gemcitabine Resistance in Pancreatic Cancer. Front Oncol. 2021;11:696371.

364. Wang M, Liu J, Zhao Y, et al. Upregulation of METTL14 mediates the elevation of PERP mRNA N(6) adenosine methylation promoting the growth and metastasis of pancreatic cancer. Mol Cancer. 2020;19(1):130.

365. Kong F, Liu X, Zhou Y, et al. Downregulation of METTL14 increases apoptosis and autophagy induced by cisplatin in pancreatic cancer cells. Int J Biochem Cell Biol. 2020;122:105731.

366. Chen S, Yang C, Wang ZW, et al. CLK1/SRSF5 pathway induces aberrant exon skipping of METTL14 and Cyclin L2 and promotes growth and metastasis of pancreatic cancer. J Hematol Oncol. 2021;14(1):60.

367. Deng J, Zhang J, Ye Y, et al. N6-methyladenosine-mediated upregulation of WTAPP1 promotes WTAP translation and Wnt signaling to facilitate pancreatic cancer progression. Cancer Res. 2021.

368. Guo X, Li K, Jiang W, et al. RNA demethylase ALKBH5 prevents pancreatic cancer progression by posttranscriptional activation of PER1 in an m6A-YTHDF2-dependent manner. Mol Cancer. 2020;19(1):91.

369. Xu X, Yu Y, Zong K, et al. Up-regulation of IGF2BP2 by multiple mechanisms in pancreatic cancer promotes cancer proliferation by activating the PI3K/Akt signaling pathway. J Exp Clin Cancer Res. 2019;38(1):497.

370. Dahlem C, Barghash A, Puchas P, et al. The Insulin-Like Growth Factor 2 mRNA Binding Protein IMP2/IGF2BP2 is Overexpressed and Correlates with Poor Survival in Pancreatic Cancer. Int J Mol Sci. 2019;20(13).

371. Taniuchi K, Furihata M, Hanazaki K, et al. IGF2BP3-mediated translation in cell protrusions promotes cell invasiveness and metastasis of pancreatic cancer. Oncotarget. 2014;5(16):6832-6845.

372. Schaeffer DF, Owen DR, Lim HJ, et al. Insulin-like growth factor 2 mRNA binding protein 3 (IGF2BP3) overexpression in pancreatic ductal adenocarcinoma correlates with poor survival. BMC Cancer. 2010;10:59.

373. Meng LD, Shi GD, Ge WL, et al. Linc01232 promotes the metastasis of pancreatic cancer by suppressing the ubiquitin-mediated degradation of HNRNPA2B1 and activating the A-Raf-induced MAPK/ERK signaling pathway. Cancer Lett. 2020;494:107-120.

374. Dai S, Zhang J, Huang S, et al. HNRNPA2B1 regulates the epithelial-mesenchymal transition in pancreatic cancer cells through the ERK/snail signalling pathway. Cancer Cell Int. 2017;17:12.

375. Chen ZY, Cai L, Zhu J, et al. Fyn requires HnRNPA2B1 and Sam68 to synergistically regulate apoptosis in pancreatic cancer. Carcinogenesis. 2011;32(10):1419-1426.

376. Barcelo C, Etchin J, Mansour MR, et al. Ribonucleoprotein HNRNPA2B1 interacts with and regulates oncogenic KRAS in pancreatic ductal adenocarcinoma cells. Gastroenterology. 2014;147(4):882-892 e888.

377. Huang XT, Li JH, Zhu XX, et al. HNRNPC impedes m(6)A-dependent anti-metastatic alternative splicing events in pancreatic ductal adenocarcinoma. Cancer Lett. 2021;518:196-206.

378. Zeng J, Zhang H, Tan Y, et al. m6A demethylase FTO suppresses pancreatic cancer tumorigenesis by demethylating PJA2 and inhibiting Wnt signaling. Mol Ther Nucleic Acids. 2021;25:277-292.

379. Tang B, Yang Y, Kang M, et al. m(6)A demethylase ALKBH5 inhibits pancreatic cancer tumorigenesis by decreasing WIF-1 RNA methylation and mediating Wnt signaling. Mol Cancer. 2020;19(1):3.

380. He Y, Hu H, Wang Y, et al. ALKBH5 Inhibits Pancreatic Cancer Motility by Decreasing Long Non-Coding RNA KCNK15-AS1 Methylation. Cell Physiol Biochem. 2018;48(2):838-846.

381. Cho SH, Ha M, Cho YH, et al. ALKBH5 gene is a novel biomarker that predicts the prognosis of pancreatic cancer: A retrospective multicohort study. Ann Hepatobiliary Pancreat Surg. 2018;22(4):305-309.

382. Hou Y, Zhang Q, Pang W, et al. YTHDC1-mediated augmentation of miR-30d in repressing pancreatic tumorigenesis via attenuation of RUNX1-induced transcriptional activation of Warburg effect. Cell Death Differ. 2021.

383. Yankova E, Blackaby W, Albertella M, et al. Small-molecule inhibition of METTL3 as a strategy against myeloid leukaemia. Nature. 2021;593(7860):597-601.

384. Vu LP, Pickering BF, Cheng Y, et al. The N(6)-methyladenosine (m(6)A)-forming enzyme METTL3 controls myeloid differentiation of normal hematopoietic and leukemia cells. Nat Med. 2017;23(11):1369-1376.

385. Sun C, Chang L, Liu C, et al. The study of METTL3 and METTL14 expressions in childhood ETV6/RUNX1-positive acute lymphoblastic leukemia. Mol Genet Genomic Med. 2019;7(10):e00933.

386. Liu X, Huang L, Huang K, et al. Novel Associations Between METTL3 Gene Polymorphisms and Pediatric Acute Lymphoblastic Leukemia: A Five-Center Case-Control Study. Front Oncol. 2021;11:635251.

387. Ianniello Z, Sorci M, Ceci Ginistrelli L, et al. New insight into the catalytic -dependent and -independent roles of METTL3 in sustaining aberrant translation in chronic myeloid leukemia. Cell Death Dis. 2021;12(10):870.

388. Pan ZP, Wang B, Hou DY, et al. METTL3 mediates bone marrow mesenchymal stem cell adipogenesis to promote chemoresistance in acute myeloid leukaemia. Febs Open Bio. 2021;11(6):1659-1672.

389. Weng H, Huang H, Wu H, et al. METTL14 Inhibits Hematopoietic Stem/Progenitor Differentiation and Promotes Leukemogenesis via mRNA m(6)A Modification. Cell Stem Cell. 2018;22(2):191-205 e199.

390. Zhang L, Khadka B, Wu J, et al. Bone Marrow Mesenchymal Stem Cells-Derived Exosomal miR-425-5p Inhibits Acute Myeloid Leukemia Cell Proliferation, Apoptosis, Invasion and Migration by Targeting WTAP. Onco Targets Ther. 2021;14:4901-4914.

391. Bansal H, Yihua Q, Iyer SP, et al. WTAP is a novel oncogenic protein in acute myeloid leukemia. Leukemia. 2014;28(5):1171-1174.

392. Wang X, Tian L, Li Y, et al. RBM15 facilitates laryngeal squamous cell carcinoma progression by regulating TMBIM6 stability through IGF2BP3 dependent. J Exp Clin Cancer Res. 2021;40(1):80.

393. Takeda A, Shimada A, Hamamoto K, et al. Detection of RBM15-MKL1 fusion was useful for diagnosis and monitoring of minimal residual disease in infant acute megakaryoblastic leukemia. Acta Med Okayama. 2014;68(2):119-123.

394. Langenberg-Ververgaert K, Renzi S, Fuligni F, et al. TERT promotor variant associated with poor clinical outcome in a patient with novel RBM15-MKL1 fusion-positive pediatric acute megakaryoblastic leukemia. Pediatr Blood Cancer. 2021;68(1):e28542.

395. Qing Y, Dong L, Gao L, et al. R-2-hydroxyglutarate attenuates aerobic glycolysis in leukemia by targeting the FTO/m(6)A/PFKP/LDHB axis. Mol Cell. 2021;81(5):922-939 e929.

396. Li Z, Weng H, Su R, et al. FTO Plays an Oncogenic Role in Acute Myeloid Leukemia as a N(6)-Methyladenosine RNA Demethylase. Cancer Cell. 2017;31(1):127-141.

397. Wang J, Li Y, Wang P, et al. Leukemogenic Chromatin Alterations Promote AML Leukemia Stem Cells via a KDM4C-ALKBH5-AXL Signaling Axis. Cell Stem Cell. 2020;27(1):81-97 e88.

398. Shen C, Sheng Y, Zhu AC, et al. RNA Demethylase ALKBH5 Selectively Promotes Tumorigenesis and Cancer Stem Cell Self-Renewal in Acute Myeloid Leukemia. Cell Stem Cell. 2020;27(1):64-80 e69.

399. Gong H, Liu L, Cui L, et al. ALKBH5-mediated m6A-demethylation of USP1 regulated T-cell acute lymphoblastic leukemia cell glucocorticoid resistance by Aurora B. Mol Carcinog. 2021;60(9):644-657.

400. Paris J, Morgan M, Campos J, et al. Targeting the RNA m(6)A Reader YTHDF2 Selectively Compromises Cancer Stem Cells in Acute Myeloid Leukemia. Cell Stem Cell. 2019;25(1):137-148 e136.

401. Chen Z, Shao YL, Wang LL, et al. YTHDF2 is a potential target of AML1/ETO-HIF1alpha loop-mediated cell proliferation in t(8;21) AML. Oncogene. 2021;40(22):3786-3798.

402. Stoskus M, Vaitkeviciene G, Eidukaite A, Griskevicius L. ETV6/RUNX1 transcript is a target of RNA-binding protein IGF2BP1 in t(12;21)(p13;q22)-positive acute lymphoblastic leukemia. Blood Cells Mol Dis. 2016;57:30-34.

403. Stoskus M, Eidukaite A, Griskevicius L. Defining the significance of IGF2BP1 overexpression in t(12;21)(p13;q22)-positive leukemia REH cells. Leuk Res. 2016;47:16-21.

404. Sharma G, Boby E, Nidhi T, et al. Diagnostic Utility of IGF2BP1 and Its Targets as Potential Biomarkers in ETV6-RUNX1 Positive B-Cell Acute Lymphoblastic Leukemia. Front Oncol. 2021;11:588101.

405. Liu C, Ma Y, Wang R, Su G. LINC00987 knockdown inhibits the progression of acute myeloid leukemia by suppressing IGF2BP2-mediated PA2G4 expression. Anticancer Drugs. 2021.

406. He X, Li W, Liang X, et al. IGF2BP2 Overexpression Indicates Poor Survival in Patients with Acute Myelocytic Leukemia. Cell Physiol Biochem. 2018;51(4):1945-1956.

407. Tran TM, Philipp J, Bassi JS, et al. The RNA-binding protein IGF2BP3 is critical for MLL-AF4-mediated leukemogenesis. Leukemia. 2021.

408. Makinen A, Nikkila A, Haapaniemi T, et al. IGF2BP3 Associates with Proliferative Phenotype and Prognostic Features in B-Cell Acute Lymphoblastic Leukemia. Cancers (Basel). 2021;13(7).

409. He L, Chen S, Ying Y, et al. MicroRNA-501-3p inhibits the proliferation of kidney cancer cells by targeting WTAP. Cancer Med. 2021.

410. Jiang Y, Zhang H, Li W, et al. LINC01426 contributes to clear cell renal cell carcinoma progression by modulating CTBP1/miR-423-5p/FOXM1 axis via interacting with IGF2BP1. J Cell Physiol. 2021;236(1):427-439.

411. Huang X, Huang M, Kong L, Li Y. miR-372 suppresses tumour proliferation and invasion by targeting IGF2BP1 in renal cell carcinoma. Cell Prolif. 2015;48(5):593-599.

412. Xie X, Lin J, Fan X, et al. LncRNA CDKN2B-AS1 stabilized by IGF2BP3 drives the malignancy of renal clear cell carcinoma through epigenetically activating NUF2 transcription. Cell Death Dis. 2021;12(2):201.

413. Gu Y, Niu S, Wang Y, et al. DMDRMR-Mediated Regulation of m(6)A-Modified CDK4 by m(6)A Reader IGF2BP3 Drives ccRCC Progression. Cancer Res. 2021;81(4):923-934.

414. Liu Y, Zhang H, Li X, et al. Identification of anti-tumoral feedback loop between VHLalpha and hnRNPA2B1 in renal cancer. Cell Death Dis. 2020;11(8):688.

415. Zhang C, Chen L, Liu Y, et al. Downregulated METTL14 accumulates BPTF that reinforces super-enhancers and distal lung metastasis via glycolytic reprogramming in renal cell carcinoma. Theranostics. 2021;11(8):3676-3693.

416. Xu T, Gao S, Ruan H, et al. METTL14 Acts as a Potential Regulator of Tumor Immune and Progression in Clear Cell Renal Cell Carcinoma. Front Genet. 2021;12:609174.

417. Wang Y, Cong R, Liu S, et al. Decreased expression of METTL14 predicts poor prognosis and construction of a prognostic signature for clear cell renal cell carcinoma. Cancer Cell Int. 2021;21(1):46.

418. Wang Q, Zhang H, Chen Q, et al. Identification of METTL14 in Kidney Renal Clear Cell Carcinoma Using Bioinformatics Analysis. Dis Markers. 2019;2019:5648783.

419. Zhuang C, Zhuang C, Luo X, et al. N6-methyladenosine demethylase FTO suppresses clear cell renal cell carcinoma through a novel FTO-PGC-1alpha signalling axis. J Cell Mol Med. 2019;23(3):2163-2173.

420. Zhao J, Lu L. Interplay between RNA Methylation EraserFTO and Writer METTL3in Renal Clear Cell Carcinoma Patient Survival. Recent Pat Anticancer Drug Discov. 2021.

421. Zhang C, Chen L, Lou W, et al. Aberrant activation of m6A demethylase FTO renders HIF2alpha(low/-) clear cell renal cell carcinoma sensitive to BRD9 inhibitors. Sci Transl Med. 2021;13(613):eabf6045.

422. Strick A, von Hagen F, Gundert L, et al. The N(6) -methyladenosine (m(6) A) erasers alkylation repair homologue 5 (ALKBH5) and fat mass and obesity-associated protein (FTO) are prognostic biomarkers in patients with clear cell renal carcinoma. BJU Int. 2020;125(4):617-624.

423. Su G, Liu T, Han X, et al. YTHDF2 is a Potential Biomarker and Associated with Immune Infiltration in Kidney Renal Clear Cell Carcinoma. Front Pharmacol. 2021;12:709548.

424. Mu Z, Dong D, Sun M, et al. Prognostic Value of YTHDF2 in Clear Cell Renal Cell Carcinoma. Front Oncol. 2020;10:1566.

425. Zhang X, Wang F, Wang Z, et al. ALKBH5 promotes the proliferation of renal cell carcinoma by regulating AURKB expression in an m(6)A-dependent manner. Ann Transl Med. 2020;8(10):646.

426. Wu H, Xu H, Jia D, et al. METTL3-induced UCK2 m(6)A hypermethylation promotes melanoma cancer cell metastasis via the WNT/beta-catenin pathway. Ann Transl Med. 2021;9(14):1155.

427. Chang X, Lin YY, Bai LN, Zhu W. miR-302a-3p suppresses melanoma cell progression via targeting METTL3. J Chemother. 2021:1-12.

428. Bhattarai PY, Kim G, Poudel M, et al. METTL3 induces PLX4032 resistance in melanoma by promoting m(6)A-dependent EGFR translation. Cancer Lett. 2021;522:44-56.

429. Yang S, Wei J, Cui YH, et al. m(6)A mRNA demethylase FTO regulates melanoma tumorigenicity and response to anti-PD-1 blockade. Nat Commun. 2019;10(1):2782.

430. Hao L, Yin J, Yang H, et al. ALKBH5-mediated m(6)A demethylation of FOXM1 mRNA promotes progression of uveal melanoma. Aging (Albany NY). 2021;13(3):4045-4062.

431. Chen R, Zhang X, Wang C. LncRNA HOXB-AS1 promotes cell growth in multiple myeloma via FUT4 mRNA stability by ELAVL1. J Cell Biochem. 2020;121(10):4043-4051.

432. Li T, Gu M, Deng A, Qian C. Increased expression of YTHDF1 and HNRNPA2B1 as potent biomarkers for melanoma: a systematic analysis. Cancer Cell Int. 2020;20:239.

433. Yu J, Chai P, Xie M, et al. Histone lactylation drives oncogenesis by facilitating m(6)A reader protein YTHDF2 expression in ocular melanoma. Genome Biol. 2021;22(1):85.

434. Kim T, Havighurst T, Kim K, et al. Targeting insulin-like growth factor 2 mRNA-binding protein 1 (IGF2BP1) in metastatic melanoma to increase efficacy of BRAF(V600E) inhibitors. Mol Carcinog. 2018;57(5):678-683.

435. Ghoshal A, Rodrigues LC, Gowda CP, et al. Extracellular vesicle-dependent effect of RNA-binding protein IGF2BP1 on melanoma metastasis. Oncogene. 2019;38(21):4182-4196.

436. Hanniford D, Ulloa-Morales A, Karz A, et al. Epigenetic Silencing of CDR1as Drives IGF2BP3-Mediated Melanoma Invasion and Metastasis. Cancer Cell. 2020;37(1):55-70 e15.

437. Jiang F, Tang X, Tang C, et al. HNRNPA2B1 promotes multiple myeloma progression by increasing AKT3 expression via m6A-dependent stabilization of ILF3 mRNA. J Hematol Oncol. 2021;14(1):54.

438. Ai Y, Liu S, Luo H, et al. METTL3 Intensifies the Progress of Oral Squamous Cell Carcinoma via Modulating the m6A Amount of PRMT5 and PD-L1. J Immunol Res. 2021;2021:6149558.

439. Liu ZF, Yang J, Wei SP, et al. Upregulated METTL3 in nasopharyngeal carcinoma enhances the motility of cancer cells. Kaohsiung J Med Sci. 2020;36(11):895-903.

440. Li X, Xie X, Gu Y, et al. Fat mass and obesity-associated protein regulates tumorigenesis of arecoline-promoted human oral carcinoma. Cancer Med. 2021;10(18):6402-6415.

441. Shriwas O, Priyadarshini M, Samal SK, et al. DDX3 modulates cisplatin resistance in OSCC through ALKBH5-mediated m(6)A-demethylation of FOXM1 and NANOG. Apoptosis. 2020;25(3-4):233-246.

442. Hu W, Li H, Wang S. LncRNA SNHG7 promotes the proliferation of nasopharyngeal carcinoma by miR-514a-5p/ELAVL1 axis. BMC Cancer. 2020;20(1):376.

443. Zhao W, Cui Y, Liu L, et al. METTL3 Facilitates Oral Squamous Cell Carcinoma Tumorigenesis by Enhancing c-Myc Stability via YTHDF1-Mediated m(6)A Modification. Mol Ther Nucleic Acids. 2020;20:1-12.

444. Yang H, Fu G, Liu F, et al. LncRNA THOR promotes tongue squamous cell carcinomas by stabilizing IGF2BP1 downstream targets. Biochimie. 2019;165:9-18.

445. Qiu L, Zheng L, Gan C, et al. circBICD2 targets miR-149-5p/IGF2BP1 axis to regulate oral squamous cell carcinoma progression. J Oral Pathol Med. 2021;50(7):668-680.

446. Wang SS, Lv Y, Xu XC, et al. Triptonide inhibits human nasopharyngeal carcinoma cell growth via disrupting Lnc-RNA THOR-IGF2BP1 signaling. Cancer Lett. 2019;443:13-24.

447. Deng X, Jiang Q, Liu Z, Chen W. Clinical Significance of an m6A Reader Gene, IGF2BP2, in Head and Neck Squamous Cell Carcinoma. Front Mol Biosci. 2020;7:68.

448. Leng F, Miu YY, Zhang Y, et al. A micro-peptide encoded by HOXB-AS3 promotes the proliferation and viability of oral squamous cell carcinoma cell lines by directly binding with IGF2BP2 to stabilize c-Myc. Oncol Lett. 2021;22(4):697.

449. Chou CH, Chang CY, Lu HJ, et al. IGF2BP2 Polymorphisms Are Associated with Clinical Characteristics and Development of Oral Cancer. Int J Mol Sci. 2020;21(16).

450. Ma Y, Jin Y, Li C, et al. LncRNA MSC-AS1 motivates the development of melanoma by binding to miR-302a-3p and recruiting IGF2BP2 to elevate LEF1 expression. Exp Dermatol. 2021.

451. Wu K, Wang X, Yu H, et al. LINC00460 facilitated tongue squamous cell carcinoma progression via the miR-320b/IGF2BP3 axis. Oral Dis. 2021.

452. Liu J, Jiang X, Zou A, et al. circIGHG-Induced Epithelial-to-Mesenchymal Transition Promotes Oral Squamous Cell Carcinoma Progression via miR-142-5p/IGF2BP3 Signaling. Cancer Res. 2021;81(2):344-355.

453. Gupta A, Yadav S, Pt A, et al. The HNRNPA2B1-MST1R-Akt axis contributes to epithelial-to-mesenchymal transition in head and neck cancer. Lab Invest. 2020;100(12):1589-1601.

454. Zhang E, Li X. LncRNA SOX2-OT regulates proliferation and metastasis of nasopharyngeal carcinoma cells through miR-146b-5p/HNRNPA2B1 pathway. J Cell Biochem. 2019;120(10):16575-16588.

455. Huang GZ, Wu QQ, Zheng ZN, et al. M6A-related bioinformatics analysis reveals that HNRNPC facilitates progression of OSCC via EMT. Aging (Albany NY). 2020;12(12):11667-11684.

456. Li Y, Zheng JN, Wang EH, et al. The m6A reader protein YTHDC2 is a potential biomarker and associated with immune infiltration in head and neck squamous cell carcinoma. Peerj. 2020;8:e10385.

457. He JJ, Li Z, Rong ZX, et al. m(6)A Reader YTHDC2 Promotes Radiotherapy Resistance of Nasopharyngeal Carcinoma via Activating IGF1R/AKT/S6 Signaling Axis. Front Oncol. 2020;10:1166.

458. Shi J, Chen G, Dong X, et al. METTL3 Promotes the Resistance of Glioma to Temozolomide via Increasing MGMT and ANPG in a m(6)A Dependent Manner. Front Oncol. 2021;11:702983.

459. Han J, Du S, Wu C, et al. METTL3 participates in glioma development by regulating the methylation level of COL4A1. J BUON. 2021;26(4):1556-1562.

460. Chang YZ, Chai RC, Pang B, et al. METTL3 enhances the stability of MALAT1 with the assistance of HuR via m6A modification and activates NF-kappaB to promote the malignant progression of IDH-wildtype glioma. Cancer Lett. 2021;511:36-46.

461. Chai RC, Chang YZ, Chang X, et al. YTHDF2 facilitates UBXN1 mRNA decay by recognizing METTL3-mediated m(6)A modification to activate NF-kappaB and promote the malignant progression of glioma. J Hematol Oncol. 2021;14(1):109.

462. Mao XG, Yan M, Xue XY, et al. Overexpression of ZNF217 in glioblastoma contributes to the maintenance of glioma stem cells regulated by hypoxia-inducible factors. Lab Invest. 2011;91(7):1068-1078.

463. Zhang S, Zhao BS, Zhou A, et al. m(6)A Demethylase ALKBH5 Maintains Tumorigenicity of Glioblastoma Stem-like Cells by Sustaining FOXM1 Expression and Cell Proliferation Program. Cancer Cell. 2017;31(4):591-606 e596.

464. Liu Z, Chen Y, Wang L, Ji S. ALKBH5 Promotes the Proliferation of Glioma Cells via Enhancing the mRNA Stability of G6PD. Neurochem Res. 2021;46(11):3003-3011.

465. Liu B, Zhou J, Wang C, et al. LncRNA SOX2OT promotes temozolomide resistance by elevating SOX2 expression via ALKBH5-mediated epigenetic regulation in glioblastoma. Cell Death Dis. 2020;11(5):384.

466. Kowalski-Chauvel A, Lacore MG, Arnauduc F, et al. The m6A RNA Demethylase ALKBH5 Promotes Radioresistance and Invasion Capability of Glioma Stem Cells. Cancers (Basel). 2020;13(1).

467. Cui Y, Wang Q, Lin J, et al. miRNA-193a-3p Regulates the AKT2 Pathway to Inhibit the Growth and Promote the Apoptosis of Glioma Cells by Targeting ALKBH5. Front Oncol. 2021;11:600451.

468. Yarmishyn AA, Yang YP, Lu KH, et al. Musashi-1 promotes cancer stem cell properties of glioblastoma cells via upregulation of YTHDF1. Cancer Cell Int. 2020;20(1):597.

469. Xu C, Yuan B, He T, et al. Prognostic values of YTHDF1 regulated negatively by mir-3436 in Glioma. J Cell Mol Med. 2020;24(13):7538-7549.

470. Lin X, Wang Z, Yang G, et al. YTHDF2 correlates with tumor immune infiltrates in lower-grade glioma. Aging (Albany NY). 2020;12(18):18476-18500.

471. Fang R, Chen X, Zhang S, et al. EGFR/SRC/ERK-stabilized YTHDF2 promotes cholesterol dysregulation and invasive growth of glioblastoma. Nat Commun. 2021;12(1):177.

472. Dixit D, Prager BC, Gimple RC, et al. The RNA m6A Reader YTHDF2 Maintains Oncogene Expression and Is a Targetable Dependency in Glioblastoma Stem Cells. Cancer Discov. 2021;11(2):480-499.

473. Zhan WL, Gao N, Tu GL, et al. LncRNA LINC00689 Promotes the Tumorigenesis of Glioma via Mediation of miR-526b-3p/IGF2BP1 Axis. Neuromolecular Med. 2021;23(3):383-394.

474. Wang RJ, Li JW, Bao BH, et al. MicroRNA-873 (miRNA-873) inhibits glioblastoma tumorigenesis and metastasis by suppressing the expression of IGF2BP1. J Biol Chem. 2015;290(14):8938-8948.

475. Luo Y, Sun R, Zhang J, et al. miR-506 inhibits the proliferation and invasion by targeting IGF2BP1 in glioblastoma. Am J Transl Res. 2015;7(10):2007-2014.

476. Liu P, Zhao P, Li B, et al. LncRNA PCAT6 Regulated by YY1 Accelerates the Progression of Glioblastoma via miR-513/IGF2BP1. Neurochem Res. 2020;45(12):2894-2902.

477. Li ZW, Xue M, Zhu BX, et al. microRNA-4500 inhibits human glioma cell progression by targeting IGF2BP1. Biochem Biophys Res Commun. 2019;513(4):800-806.

478. Yang Y, Liu X, Cheng L, et al. Tumor Suppressor microRNA-138 Suppresses Low-Grade Glioma Development and Metastasis via Regulating IGF2BP2. Onco Targets Ther. 2020;13:2247-2260.

479. Wang X, Li X, Zhou Y, et al. Long non-coding RNA OIP5-AS1 inhibition upregulates microRNA-129-5p to repress resistance to temozolomide in glioblastoma cells via downregulating IGF2BP2. Cell Biol Toxicol. 2021.

480. Li H, Wang D, Yi B, et al. SUMOylation of IGF2BP2 promotes vasculogenic mimicry of glioma via regulating OIP5-AS1/miR-495-3p axis. Int J Biol Sci. 2021;17(11):2912-2930.

481. Ding L, Wang L, Guo F. microRNA188 acts as a tumour suppressor in glioma by directly targeting the IGF2BP2 gene. Mol Med Rep. 2017;16(5):7124-7130.

482. Kouhkan F, Mobarra N, Soufi-Zomorrod M, et al. MicroRNA-129-1 acts as tumour suppressor and induces cell cycle arrest of GBM cancer cells through targeting IGF2BP3 and MAPK1. J Med Genet. 2016;53(1):24-33.

483. Jin P, Huang Y, Zhu P, et al. CircRNA circHIPK3 serves as a prognostic marker to promote glioma progression by regulating miR-654/IGF2BP3 signaling. Biochem Biophys Res Commun. 2018;503(3):1570-1574.

484. Liu Z, Liu N, Huang Z, Wang W. METTL14 Overexpression Promotes Osteosarcoma Cell Apoptosis and Slows Tumor Progression via Caspase 3 Activation. Cancer Manag Res. 2020;12:12759-12767.

485. Smeester BA, Draper GM, Slipek NJ, et al. Implication of ZNF217 in Accelerating Tumor Development and Therapeutically Targeting ZNF217-Induced PI3K-AKT Signaling for the Treatment of Metastatic Osteosarcoma. Mol Cancer Ther. 2020;19(12):2528-2541.

486. Ling Z, Chen L, Zhao J. m6A-dependent up-regulation of DRG1 by METTL3 and ELAVL1 promotes growth, migration, and colony formation in osteosarcoma. Biosci Rep. 2020;40(4).

487. Wang L, Aireti A, Aihaiti A, Li K. Expression of microRNA-150 and its Target Gene IGF2BP1 in Human Osteosarcoma and their Clinical Implications. Pathol Oncol Res. 2019;25(2):527-533.

488. Qu Y, Pan S, Kang M, et al. MicroRNA-150 functions as a tumor suppressor in osteosarcoma by targeting IGF2BP1. Tumour Biol. 2016;37(4):5275-5284.

489. Yang J, Han Q, Li C, et al. Circular RNA circ_0001105 Inhibits Progression and Metastasis of Osteosarcoma by Sponging miR-766 and Activating YTHDF2 Expression. Onco Targets Ther. 2020;13:1723-1736.

490. Yuan Y, Yan G, He M, et al. ALKBH5 suppresses tumor progression via an m(6)A-dependent epigenetic silencing of pre-miR-181b-1/YAP signaling axis in osteosarcoma. Cell Death Dis. 2021;12(1):60.

491. Chen S, Zhou L, Wang Y. ALKBH5-mediated m(6)A demethylation of lncRNA PVT1 plays an oncogenic role in osteosarcoma. Cancer Cell Int. 2020;20:34.

492. Lixin S, Wei S, Haibin S, et al. miR-885-5p inhibits proliferation and metastasis by targeting IGF2BP1 and GALNT3 in human intrahepatic cholangiocarcinoma. Mol Carcinog. 2020;59(12):1371-1381.

493. Kessler SM, Lederer E, Laggai S, et al. IMP2/IGF2BP2 expression, but not IMP1 and IMP3, predicts poor outcome in patients and high tumor growth rate in xenograft models of gallbladder cancer. Oncotarget. 2017;8(52):89736-89745.

494. Rong ZX, Li Z, He JJ, et al. Downregulation of Fat Mass and Obesity Associated (FTO) Promotes the Progression of Intrahepatic Cholangiocarcinoma. Front Oncol. 2019;9:369.

495. Qiu X, Yang S, Wang S, et al. M(6)A Demethylase ALKBH5 Regulates PD-L1 Expression and Tumor Immunoenvironment in Intrahepatic Cholangiocarcinoma. Cancer Res. 2021;81(18):4778-4793.

496. Shang Y. LncRNA THOR acts as a retinoblastoma promoter through enhancing the combination of c-myc mRNA and IGF2BP1 protein. Biomed Pharmacother. 2018;106:1243-1249.

497. Zhao H, Dong S, Du J, et al. Analysis of miRNA-mRNA Crosstalk in Radiation-Induced Mouse Thymic Lymphomas to Identify miR-486 as a Critical Regulator by Targeting IGF2BP3 mRNA. Front Oncol. 2020;10:574001.

498. Faye MD, Beug ST, Graber TE, et al. IGF2BP1 controls cell death and drug resistance in rhabdomyosarcomas by regulating translation of cIAP1. Oncogene. 2015;34(12):1532-1541.

499. Chen H, Xiang Y, Yin Y, et al. The m6A methyltransferase METTL3 regulates autophagy and sensitivity to cisplatin by targeting ATG5 in seminoma. Transl Androl Urol. 2021;10(4):1711-1722.

500. Iaiza A, Tito C, Ianniello Z, et al. METTL3-dependent MALAT1 delocalization drives c-Myc induction in thymic epithelial tumors. Clin Epigenetics. 2021;13(1):173.
